# Irreversible HER2 inhibitors overcome resistance to the RSL3 ferroptosis inducer in non-HER2 amplified luminal breast cancer

**DOI:** 10.1038/s41419-023-06042-1

**Published:** 2023-08-18

**Authors:** Soon Young Park, Kang Jin Jeong, Alfonso Poire, Dong Zhang, Yiu Huen Tsang, Aurora S. Blucher, Gordon B. Mills

**Affiliations:** Division of Oncologic Sciences, Knight Cancer Institute, Oregon Health Sciences University, Portland, OR USA

**Keywords:** Cell death, Target identification

## Abstract

Ferroptosis, a form of programed cell death, can be promoted by inhibitors of the xCT transporter (erastin) or GPX4 (RSL3). We found that GPX4, but not the xCT transporter, is selectively elevated in luminal breast cancer. Consistent with this observation, the majority of luminal breast cancer cell lines are exquisitely sensitive to RSL3 with limited sensitivity to erastin. In RSL3-resistant, but not sensitive, luminal breast cancer cell lines, RSL3 induces HER2 pathway activation. Irreversible HER2 inhibitors including neratinib reversed RSL3 resistance in constitutively RSL3-resistant cell lines. Combination treatment with RSL3 and neratinib increases ferroptosis through mitochondrial iron-dependent reactive oxygen species production and lipid peroxidation. RSL3 also activated replication stress and concomitant S phase and G2/M blockade leading to sensitivity to targeting the DNA damage checkpoint. Together, our data support the exploration of RSL3 combined with irreversible HER2 inhibitors in clinical trials.

## Introduction

Ferroptosis-regulated cell death represents an iron-dependent programmed cell death as a consequence of lipid peroxidation induced by reactive oxygen species (ROS) [1]. Cells undergoing ferroptosis exhibit changes in mitochondrial morphology and crista structure without the nuclear condensation or chromatin margination observed in apoptosis [2]. Cystine is imported through xCT (SLC7A11 and SLC3A2) transmembrane antiporters with xCT inhibitors such as erastin or sulfasalazine decreasing cystine uptake, which is required for glutathione (GSH) synthesis [2]. Depletion of GSH can lead to an accumulation of reactive oxygen species (ROS), increased lipid peroxidation, and ultimately ferroptotic cell death [3]. Glutathione peroxidase 4 (GPX4), an enzyme responsible for reducing lipid peroxides induced by ROS, plays a critical role in cellular antioxidant defense systems and thus in ferroptosis [4].

Ductal breast cancer (BC), the most common form of BC, is subdivided into luminal or HR (estrogen and progesterone) positive, HER2-positive, and triple-negative breast cancer (TNBC) that lacks ER, PR or HER2 expression. In the absence of HER2-targeted therapy, HER2 + BC had the worst prognosis [5, 6]. However, new therapy approaches including antibodies against HER2 (trastuzumab and pertuzumab), reversible and irreversible HER2 enzyme inhibitors (lapatinib, neratinib (for example)), and antibody-drug conjugates (ado-trastuzumab emtansine and fam-trastuzumab-deruxtecan-nxki) have changed the life history of HER2 + BC to a much more favorable outcome [7–11]. The improved outcomes in HER2-positive tumors may be due, in part, to ferroptosis as neratinib an irreversible HER2 inhibitor promotes ferroptosis and inhibits brain metastasis in a HER2+-positive BC model [12]. Indeed, a recent study indicates that neratinib decreases xCT expression and function providing a potential mechanism [13].

Anticancer therapies including chemotherapy, targeted therapies, immunotherapy, and radiotherapy have been reported to promote ferroptosis through repression of SLC7A11 expression in multiple model systems [13–16]. SLC7A11 (xCT), SLC3A2 (xCT), and GPX4 expression have been reported to be elevated in a subset of TNBC breast cancer models predicting response to erastin (xCT inhibitor) and RSL3 (GPX4 inhibitor) [17]. GPX4 has been demonstrated to negatively regulate ferroptosis in gefitinib-resistant TNBC cells [18]. Siramesine (a lysosome-disrupting agent) synergizes with lapatinib in BC cell lines to induce ferroptosis [19]. JQ1 induced ferroptosis in BRD4 overexpressing TNBC [20] that is increased with proteasome inhibition [21]. In contrast to TNBC where extensive studies of ferroptosis have been reported, there are limited reports of luminal BC. We thus explored sensitivity to ferroptotic mediators and explored mechanisms leading to resistance to ferroptosis in luminal hormone receptor (HR)-positive BC cell lines to identify effective combination therapy approaches for this BC population. This is particularly important as more patients die of luminal BC than all other types of BC combined due to the frequency of luminal BC (https://seer.cancer.gov/statfacts/html/breast-subtypes.html). We show that the majority of luminal BC lines are exquisitely sensitive to RSL3 but, in contrast, have limited sensitivity to erastin. In RSL3-resistant luminal BC models, RSL3 increased HER2 pathway activation as an adaptive response. Importantly, irreversible inhibitors of HER2 including neratinib, afatinib, and dacomitinib were synergistic with RSL3 in RSL3-resistant HR-positive cell models through increased iron-dependent ROS, lipid peroxide production, mitochondrial dysfunction, and subsequent ferroptosis. Together these studies establish ferroptosis as a potential therapeutic opportunity in luminal BC, the HER2 pathway as a mechanism of resistance to ferroptosis, and irreversible HER2 inhibitors as an approach to overcome intrinsic resistance to ferroptosis in luminal BC.

## Results

### Ferroptosis inducer activity in a panel of luminal BC

**Fig. 1:**
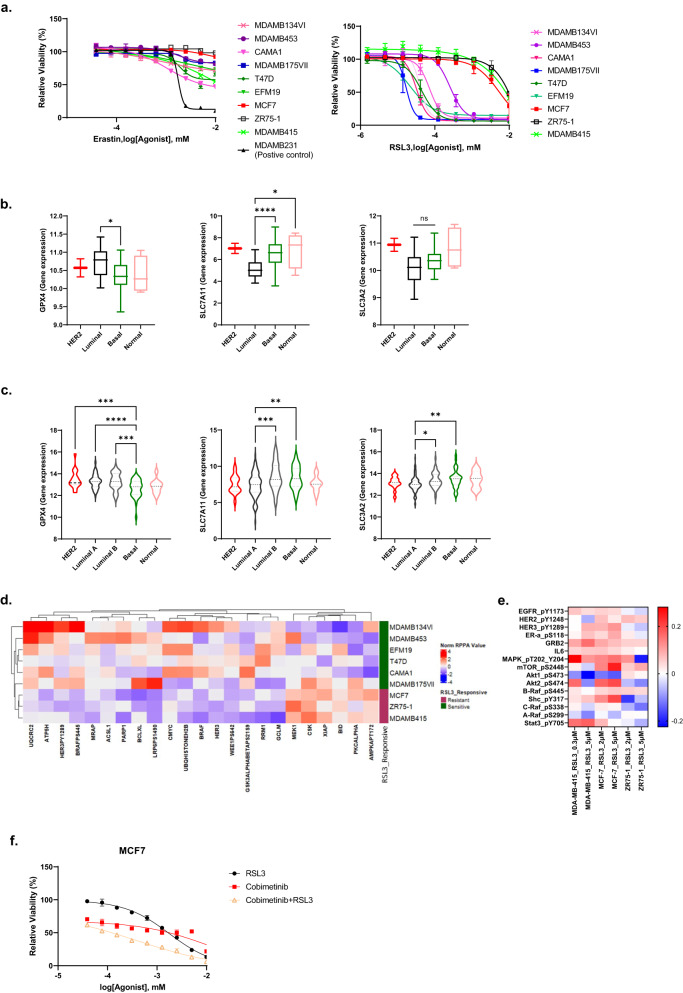
RSL3 inhibits cell growth in GPX4 expressing luminal BC. **a** The indicated luminal BC cell lines were treated with GPX4 inhibitor (RSL3) or the cystine/glutamate antiporter inhibitor (Erastin) at the indicated doses (0–10 μM) for 3 days, followed by viability assay with presto blue. MDABM231 (TNBC) was included as a positive control for Erastin activity. **b** GPX4, SLC7A11, and SLC3A2 mRNA expression across BC cell lines (**b**, HER2, *n* = 2; Luminal, *n* = 27; Basal, *n* = 20; Normal, *n* = 4). The data are presented as a box and whisker plot including the minimum value (whisker), lower quartile, median, upper quartile, and max value (whisker). *p*-values were calculated with the ANOVA test. *p < 0.05, ****p < 0.0001. **c** GPX4, SLC7A11, and SLC3A2 mRNA expression across TCGA BC cell types. The data is presented as a Violin plot including the minimum value, lower quartile, median, upper quartile, and max value. *p*-values were calculated with the ANOVA test. *p < 0.05, **p < 0.01, ***p < 0.001, ****p < 0.0001. **d** Heatmap represents the basal expression levels of proteins with significant differences based on the ANOVA test (*p* < 0.05) from RPPA data with supervised clustering based on RSL3 sensitivity. **e** Indicated cell lines were treated with RSL3 (MDAMB415: 0.3, 5 μM, MCF7 and ZR75-1: 2, 5 μM) for 3 days and changes in protein expression of HER2 family members, and targets were assessed using BIOCARTA_HER2_PATHWAY from RPPA data represented as the ratio of treated to pre-treatment. HER2 pathway signaling represents the value of the ratio of treated vs. control. **f** MCF7 cells were treated with RSL3, cobimetinib, or the combination at the indicated doses (0–10 μM) for 3 days and relative viability was determined using presto blue staining.

We assessed the activity of ferroptosis inducers in a panel of 9 luminal BC cell lines. Erastin (xCT: SLC7A11 and SLC3A2 inhibitor) had modest effects in the luminal BC cell lines with a less than 50% decrease in viability at high concentrations (Fig. 1a; Table S1). In contrast, RSL3 (GPX4 inhibitor) demonstrated marked responses in 6/9 luminal BC cell lines (Fig. 1a). However, MCF7, MDAMB415, and ZR75-1 cell lines were resistant to RSL3 with IC50 values greater than 2 µM (Fig. 1a; Table S1). The IC50 values of RSL3 in Fig. 1a were compared to the AUC values of RSL3, ML210, and ML162 GPX4 inhibitors in CTRPv2, revealing correlation coefficients of 0.83, 0.7, and 0.79, respectively (Supplementary Fig. 1a).

The expression of the targets of RSL3 and erastin, GPX4 and xCT, respectively, was investigated in BC cell lines (Fig. 1b) and patient samples (Fig. 1c). Interestingly in both cell lines and patient samples, the mRNA expression of GPX4, the target of RSL3, was elevated in luminal compared to basal BC. In contrast, the mRNA expression of xCT (SLC7A11 and SLC3A2) transporters was lower in luminal BC compared to basal BC. Protein levels of GPX4, SLC7A11, and SLC3A2 in four luminal breast cancer cell lines (MDAMB415, ZR75-1, MCF7, and CAMA1), four basal breast cancer cell lines (MDAMB436, MDAMB231, HCC1937, and HCC1806), and one normal breast epithelial cell line (MCF10A) were concordant with mRNA expression (Supplementary Fig. 1b, c). The elevated GPX4 levels could contribute to the response of luminal BC cell lines to RSL3.

We subsequently explored potential mechanisms that could contribute to the differential sensitivity of luminal BC cell lines to RSL3. Consistent with the response to RSL3 being due to susceptibility to ferroptosis, a ferroptosis-related gene-set (https://www.gsea-msigdb.org/gsea/msigdb/human/geneset/WP_FERROPTOSIS.html?keywords=Ferroptosis) was markedly elevated in RSL3-sensitive but not in RSL3-resistant luminal BC cell lines (Supplementary Fig. 1d) with a false discovery rate (FDR of (0.099)) likely due to the small number of samples in the dataset. The presence of a ferroptosis-related gene-set in the basal state in RSL3-sensitive lines suggests that RSL3-sensitive lines have a predilection to activation of the ferroptosis pathway. Using reverse phase protein arrays with 486 antibodies, we assessed baseline proteomes that could contribute to sensitivity and resistance to RSL3 (Fig. 1d). Significant differences between RSL3-sensitive and RSL3-resistant cell lines included increases in oxphos-related protein expression (UQCRC2, ATP5H) in the sensitive lines, phosphorylation of HER3 and BRAF and total BRAF levels in the sensitive lines and MEK1, CSK, XIAP, PKCalpha, BID, and phosphoAMPK in the resistant cell lines (Fig. 1d). The higher basal expression of oxphos mediators could contribute to the intrinsic sensitivity of the lines to RSL3. This suggested that the RSL3-resistant cell lines might have intrinsic resistance to ferroptosis due to decreased levels of the oxphos mediators or that they acquire a resistance phenotype upon treatment with RSL3. Indeed, tumor cells rewire signaling pathways in response to targeted therapies resulting in adaptive resistance that is frequently therapeutically tractable [22]. Strikingly, treatment of RSL3-resistant luminal BC cell lines with RSL3 resulted in a marked increase in targets associated with the BIOCARTA_HER2_PATHWAY gene-set (https://www.gsea-msigdb.org/gsea/msigdb/human/geneset/BIOCARTA_HER2_PATHWAY.html?keywords=HER2). RSL3 treatment of RSL3-resistant cell lines led to phosphorylation of the EGFR/HER2/HER3 family of receptors, as well as more variable and potentially cell line-specific changes in the phosphorylation of downstream pathway mediators including components of the RAS/MAPK and PI3K/AKT pathways (Fig. 1e). Targeting the RAS/MAPK pathway with cobimetinib, an inhibitor of MEK, in combination with RSL3 enhanced cell death in RSL3-resistant cell lines (Fig. 1f; Table S2).

### RSL3 induced HER2 signaling leads to RSL3 resistance

**Fig. 2:**
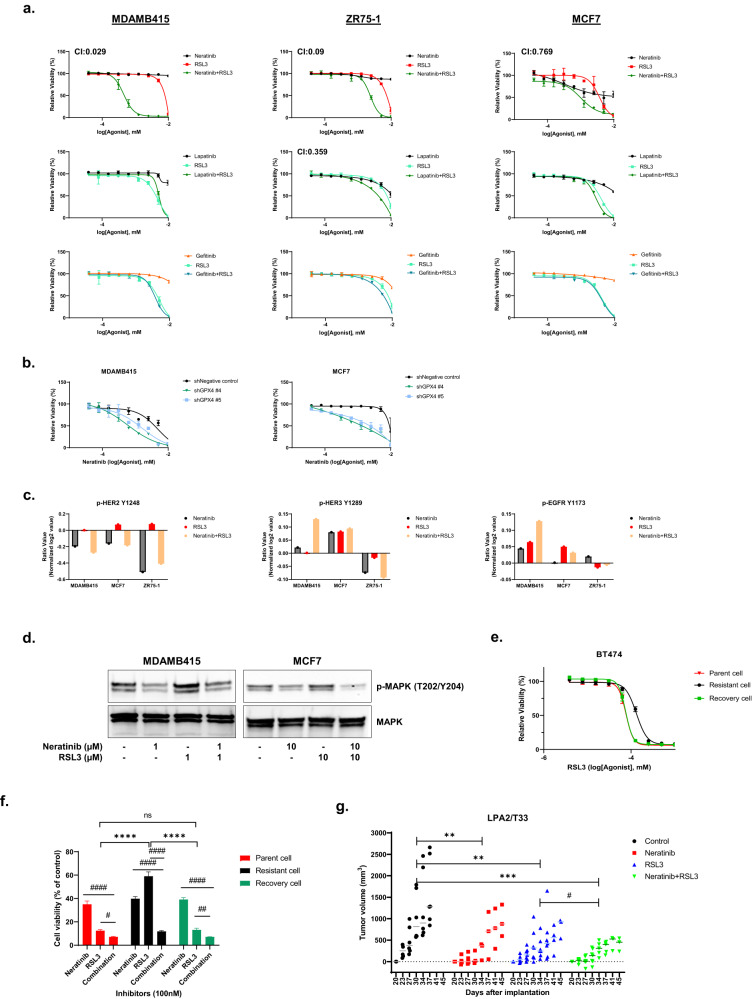
Neratinib and RSL3 combination inhibits cell growth and induces cell death in RSL3-resistant luminal BC cell lines. **a** Luminal BC cell lines were treated with RSL3, neratinib, lapatinib, or gefitinib individually or in combinations at the indicated doses (0–10 μM) for 3 days and relative viability was determined with presto blue. CI values of combination treatment were calculated using the Chou-Talalay equation with CI < 1 indicating synergy between the inhibitors. **b** MCF7 or MDAMB415 cells were stably transfected with two different GPX4 shRNA and treated with the indicated doses (0–10 μM) of neratinib for 3 days and cell viability was assessed with presto blue. **c** Cells were treated with neratinib, RSL3, or combination (MDAMB415: 0.3 μM, MCF7 and ZR75-1: 2 μM for mono and combination therapy) for 3 days and assessed by RPPA. The doses used were the IC75 values for the combination from Fig. 2a. Phosphoproteins are presented as the ratio of treatment vs. control. **d** Cells were incubated with neratinib, RSL3, or combination for 3 days. Cells were harvested, lysed, and analyzed for phosphorylated MAPK and MAPK protein levels using western blotting. **e** Parent (BT474) cells, RSL3-resistant cells (cultured with RSL3), and recovery cells (resistant cells cultured without RSL3) were treated with 10 serial doses (0–1 μM) of RSL3 for 3 days, and cell viability was assessed using presto blue. The IC50 values for parent cells were 0.059 μM, resistant cells 0.101 μM, and recovery cells 0.062 μM. **f** Parent, resistant or recovery BT474 cells were incubated with neratinib (100 nM), RSL3 (100 nM), and combination (100 nM) for 3 days and cell viability was assessed using presto blue. *p*-values were calculated with Student *t*-test with #*p* < 0.05, ##*p* < 0.01, ####*p* < 0.0001 compared with indicated treatment, ****p < 0.0001 compared with RSL3. **g** LPA2/T33 tumor fragments were inserted into the fat pad of FVB/NJ mice and allowed to establish for 20 days. The mice were randomized to 4 groups and treated with RSL3 (30 mg/kg twice weekly), neratinib (3 mg/kg daily), or the combination (N + R) for 25 days. Each data point represents an individual measurement in a mouse. *p*-values were calculated with two-way ANOVA with multiple comparisons test. **p < 0.01, ***p < 0.001 compared with control, #*p* < 0.05 compared with RSL3.

The observed increase in phosphoHER2/HER3/EGFR on the treatment of RSL3-resistant cell lines with RSL3 suggested that the adaptive upregulation of HER2 family signaling could mediate resistance to RSL3. We thus assessed the effects of EGFR family inhibitors on the response to RSL3 in the RSL3-resistant cell lines. Strikingly, the combination of the irreversible HER2 inhibitor neratinib and RSL3 demonstrated marked synergy in the MDAMB415 and ZR75-1 cell lines with a more modest interaction in the MCF7 cell line (Fig. 2a; Table S3). The synergy with neratinib was recapitulated with the irreversible inhibitors afatinib and dacomitinib but not by the reversible HER2 inhibitors like lapatinib, sapitinib, and tucatinib or with the EGFR inhibitor gefitinib (Fig. 2a; Supplementary Fig. 2a and Table S4). The synergy between neratinib and RSL3 were not observed with neratinib and erastin (Supplementary Fig. 2b and Table S5). HER2 inhibitors such as antibodies (T-DM1 and Trastuzumab) have limited activity in MCF7 cells that are not amplified for HER2 (Supplementary Fig. 2c). Furthermore, the combination of RSL3 with T-DM1 or Trastuzumab is not more active than RSL3 alone. Thus, T-DM1 or Trastuzumab do not mimic the effects of neratinib in combination with RSL3. Resistance to neratinib has been linked previously to elevated levels of xCT (SLC3A2) and increased xCT activity [13]. However, in the model systems we studies, treatment with either neratinib or RSL3 alone, as well as their combination, did not alter xCT activity compared to control in MCF7 cells. Erastin alone was sufficient to decrease xCT activity validating the assay (Supplementary Fig. 2d). The synergy with neratinib and RSL3 was recapitulated with knockdown of GPX4 with two separate shRNAs (Fig. 2b, Table S6 and see Supplementary Fig. 2e for knockdown), as well as with a different GPX4 inhibitor ML210 (Supplementary Fig. 2f; Table S7). The decrease in relative cell viability induced by the combination of RSL3 and neratinib was, at least in part, due to cell death as indicated by an increase in 7AAD staining (Supplementary Fig. 2g). Together the data support the contention that adaptive HER2 signaling mediates resistance to GPX4 inhibitors and that the resistance can be reversed by irreversible HER2 inhibitors. The role of HER2 signaling in the synergy between neratinib and RSL3 was further supported by analysis of phosphorylation of HER2 family members where phosphoHER2 was increased by RSL3 with the addition of neratinib consistently decreasing phosphoHER2 (Fig. 2c). In contrast the effect of the RSL3 and neratinib combination on phosphoHER3 and phosphoEGFR was more variable (Fig. 2c). Consistent with the decrease in HER2 phosphorylation, the combination of RSL3 and neratinib decreased phosphorylation of ERK that is located downstream in the HER2 pathway (Fig. 2d).

Over time, resistance to targeted inhibitors is frequently acquired in initially sensitive cells [22]. This can occur through the selection of cells with genomic mechanisms of resistance or alternative through persister cells. Persister cells are defined by an epigenomic resistance that is reversed on removal of the targeted therapy [23]. To investigate whether persister cells could contribute to RSL3 resistance, we generated a RSL3-resistant cell line (1.7-fold resistant to RSL3, Fig. 2e and Table S8). Consistent with persister cells contributing to RSL3 resistance, RSL3-resistant BT474 cells reverted to a RSL3-sensitive state on the removal of RSL3 (Fig. 2e). Strikingly, the RSL3-resistant BT474 cells were sensitive to the combination of RSL3 and neratinib (Fig. 2f). This suggests that the RSL3 and neratinib combination could not only reverse intrinsic RSL3 resistance but could prevent the acquisition of RSL3 resistance in sensitive cells or reverse resistance once it occurs.

Subsequently, we assessed the effectiveness of the RSL3 and neratinib combination in a syngeneic estrogen-responsive transplantable tumor model (LPA2-T33) developed from lysophosphatidic acid (LPA) receptor 2 transgenic mice [24]. LPA2-T33 tumors were modestly responsive to both RSL3 and neratinib monotherapy, with combination treatment with RSL3 and neratinib resulting in a statistically significant decrease in tumor size compared to either therapy alone (Fig. 2g). Tumor weight at the end of the study showed that combination treatment with RSL3 and neratinib significantly decreased tumor size compared with control group (Supplementary Fig. 2h). Tumor control was maintained by the combination treatment for 45 days without evidence of toxicity as assessed by monitoring animal weight (Supplementary Fig. 2i).

### RSL3 and neratinib induced cell death through ferroptosis

**Fig. 3:**
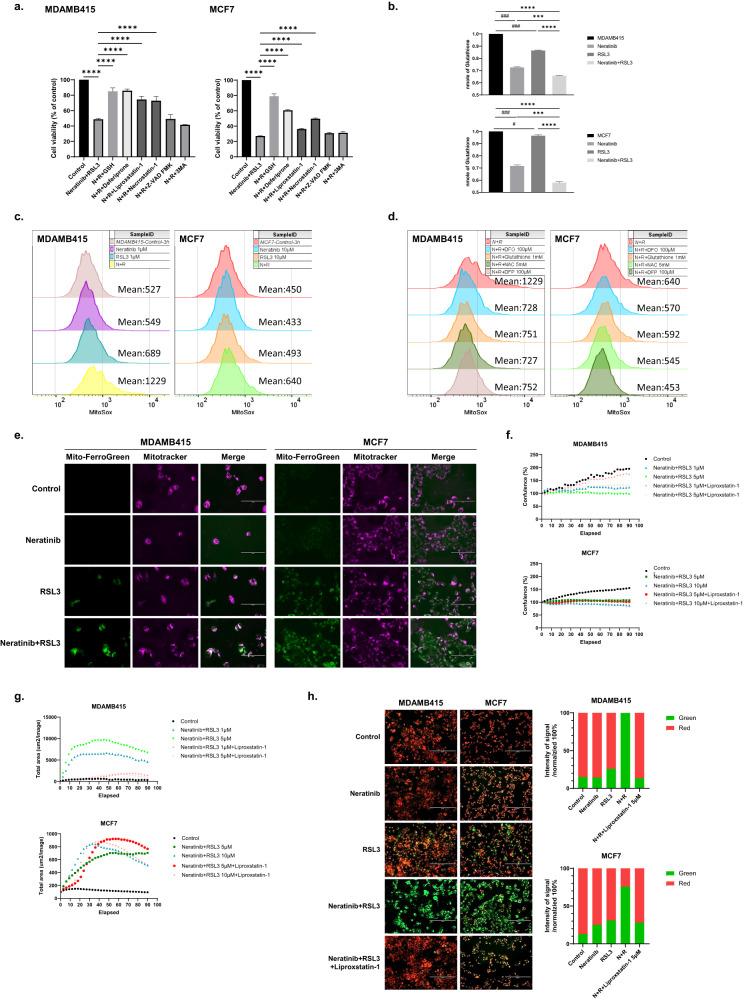
Neratinib and RSL3 induce ferroptotic cell death. **a** Cells were incubated for 72 h with neratinib and RSL3 (MDAMB415 (0.625 µM) and MCF7 (1.25 µM)) with and without the indicated inhibitors (GSH 1 mM, deferiprone 100 μM, liproxstatin-1 5 μM, necrostatin-1 25 μM, Z-VAD FMK 10 μM, 3MA 50 μM) for 72 h and cell viability assessed with presto blue. Significance was determined with a one-way ANOVA test. ****p < 0.0001. **b** MDAMB415 and MCF7 were treated with the indicated concentrations of neratinib and RSL3 (MDAMB415: 5 μM, MCF7: 10 μM) for 3 h and GSH levels were measured using a GSH assay kit (see Methods). Significance was determined with a one-way ANOVA test. #*p* < 0.05, ###*p* < 0.001 compared with control, ****p < 0.0001, ***p < 0.001 compared with the indicated treatment. **c** Cells were treated with the indicated concentrations of neratinib, RSL3 or combination (N + R) for 3 h. Mitochondrial ROS was detected by MitoSOX staining with flow cytometry analysis. **d** Cells were treated with neratinib and RSL3 (N + R, 1 µM for MDAMB435, 10 µM for MCF7). The same cells as in Fig. 3c were incubated with DFO (100 µM), GSH (1 mM), NAC (5 mM) or DFP (100 µM) for 3 h. Mitochondrial ROS was detected by MitoSOX staining with flow cytometry analysis. **e** Cells were treated with the neratinib, RSL3, or combination (N + R) for MDAMB415 (1 μM, mono and combination therapy) and MCF7 (10 μM, mono and combination therapy). Mitochondrial iron was detected with Mito-FerroGreen with co-staining with Mitotraker staining to identify mitochondria. Scale bars: 100 μm. **f** Cell confluence was monitored by IncuCyte systems every 3 h with the indicated treatments with or without liproxstatin-1 (5 µM). Images were captured from nine different ROIs, and the graph represents the mean values. **g** Total cell area of cell death (EthD1-positive staining, (red total area (μm^2^/Image)) was monitored by IncuCyte systems every 3 h with the indicated treatments with or without liproxstatin-1 (5 µM). Images were captured from nine different ROIs, and the graph represents the mean values. **h** Cells treated with neratinib and RSL3 (MDAMB415: 1 µM for mono and combination therapy, MCF7: 10 µM for mono and combination therapy) with or without liproxstatin-1 (5 µM). Lipid peroxidation was detected with BODIPY 581/591 C11 staining (oxidized, green). Scale bar: 200 μm. Imaris software was used for cell segmentation and quantification of green and red intensity per cell, which was then normalized with cell number and represented as a relative amount.

We used specific inhibitors to explore mechanisms underlying the limited activity of RSL3 in RSL3-resistant cells and the synergy induced by the neratinib and RSL3 combination. Due to the differential sensitive of MCF7 and MDAMB415 cells to RSL3 mono and combination therapy with neratinib, we used different doses to assess signaling pathways. In MDAMB415 cells, GSH, an iron chelator (deferiprone (DFP) [25] and a lipid peroxidation inhibitor (liproxstatin-1) inhibited the effects of the neratinib and RSL3 combination as did the necrosis inhibitor (necrostatin-1) [26] (Fig. 3a). In contrast a pan-caspase apoptosis inhibitor (Z-VAD-FMK) and an autophagy inhibitor 3-methyladenine (3MA) [3, 21] did not alter the growth inhibition induced by the RSL3 and neratinib combination. In MCF7 cells, GSH, deferiprone and, to a lesser extent, liproxstatin-1 and necrostatin-1 inhibited the effects of the RSL3 and neratinib combination (Fig. 3a). In ZR75-1 cells, GSH and, to a lesser degree, liproxstatin-1 reversed the effects of RSL3 and neratinib on cell viability (Supplementary Fig. 3a). The inhibition of cell death observed with GSH, deferiprone and liproxstatin-1 supports ferroptosis as a major contributor to cell death induced by the combination of RSL3 with neratinib. However, the modest inhibition by liproxstatin-1 in MCF7 cells suggests that the underlying mechanisms regulating the response to neratinib and RSL3 in MCF7 likely differ from those in MDAMB415 at some level. The effects of necrostatin-1 suggest that necroptosis either as an independent process or as a consequence of ferroptosis contributes to the activity of the RSL3 and neratinib combination.

The reversal of the effects of neratinib and RSL3 by GSH suggested that the combination could alter intracellular GSH levels. Indeed, while neratinib alone modestly decreased GSH levels, and RSL3 had minimal effects on GSH levels, the combination treatment induced a marked decrease in intracellular GSH levels in MDAMB415 and MCF7 cells (Fig. 3b).

The inhibition of the action of the RSL3 and neratinib combination by GSH and the decrease in GSH levels suggested that the combination likely increased mitochondrial ROS levels. Indeed, mitochondrial ROS levels (MitoSox) were increased slightly by RSL3 but were increased markedly by the RSL3 and neratinib combination in MDAMB415 cells and to a lesser degree in MCF7 cells (Fig. 3c). In both MDAMB415 and MCF7 cell lines, iron chelation (desferrioxamine (DFO) and DFP) as well as GSH and N-acetyl cysteine (NAC) reduced mitochondrial ROS levels consistent with these changes being associated with ferroptosis (Fig. 3d).

The effects of DFP on growth inhibition induced by the RSL3 and neratinib combination suggested that the combination could alter iron levels. As indicated by mitoferro-green staining combined with mitotracker to mark mitochondria, monotherapy with RSL3 but not neratinib induced a modest increase in mitochondrial iron (green, Fig. 3e). As predicted, the RSL3 and neratinib combination resulted in a marked increase in mitochondrial iron levels.

The changes in mitochondrial ROS and iron levels suggested that mitochondrial membrane potential could be altered by the neratinib and RSL3 combination. Indeed, as indicated by staining with JC-1, the RSL3 and neratinib combination induced a dose-dependent membrane depolarization in the RSL3-resistant cell lines (Supplementary Fig. 3b).

We next explored the apparent differential sensitivity of the effects of RSL3 and neratinib to liproxstatin-1 in MDAMB415 and MCF7 cells. In MDAMB415 cells, liproxstatin-1 (Fig. 3f), as well as another lipid peroxidase inhibitor ferrostatin-1 (Supplementary Fig. 3c), reversed the growth inhibition induced by the RSL3 and neratinib combination. Liproxstatin-1 also decreased cell death induced by the RSL3 and neratinib combination in MDAMB415 cells (Fig. 3g). However, in MCF7 cells, both inhibitors had modest effects (Fig. 3f, g, Supplementary Fig. 3c). Liproxstatin-1 reversed the growth inhibition induced by high doses of RSL3 in MDMB415 cells, consistent with its effects on growth inhibition induced by RSL3 and neratinib (Supplementary Fig. 3d). In contrast, in MCF7 cells, liproxstatin-1 did not reverse the effects of RSL3 monotherapy (Supplementary Fig. 3d) consistent once again with the effects of liproxstatin-1 on growth inhibition induced by the combination of RSL3 and neratinib. Thus, induction of liproxstatin-1-dependent lipid peroxidation may not be necessary for the completion of the ferroptosis program in MCF7 cells.

We subsequently explored the effects of the agents on lipid peroxidation. As indicated by BODIPY 581/591 C11 staining, RSL3, but not neratinib induced a modest increase in lipid peroxidation, whereas the combination of RSL3 and neratinib induced a marked increase in lipid peroxidation in both MDAMB415 and MCF7 cells (Fig. 3h). Liproxstatin-1 inhibited the lipid peroxidation induced by the RSL3 and neratinib combination albeit to a lesser degree in MCF7 cells. This suggests that the limited effects of liproxstatin-1 in MCF7 cells could be mediated by a decreased ability of liproxstatin-1 to inhibit lipid peroxidation in this cell line.

To investigate the effects of combination therapies with other irreversible HER2-targeting TKIs such as afatinib and dacomitinib in conjunction with RSL3, we assessed their impact on iron levels, reactive oxygen species (ROS), and lipid peroxidation. Combination treatment with afatinib or dacomitinib in combination with RSL3 elevated iron levels in MDAMB415 cells (Supplementary Fig. 3f). Iron levels were increased in MCF7 with monotherapy or combination therapy with afatinib or dacomitinib and RSL3. Similar to the effects on iron levels, in MCF7 cells, afatinib or dacomitinib monotherapy and combination increase ROS levels. However, in MCF7 cell lines, lipid peroxidation levels were much higher in combination treatment compared to monotherapy with afatinib and dacomitinib. At the concentrations used, afatinib and dacomitinib were less active than neratinib in terms of functional effects on iron/ROS/lipid peroxidation and effects on cell growth in the presence of RSL3 (Supplementary Fig. 2a).

Overall, the data are consistent with the combination of RSL3 and neratinib inducing a marked increase in ferroptosis in RSL3-resistant luminal BC cell lines through an iron-dependent mechanism that includes depletion of GSH, induction of mitochondrial ROS production, mitochondrial depolarization, and induction of lipid peroxidation. Why inhibition of lipid peroxidation with liproxstatin-1 or ferrostatin-1 efficiently reverses the effects of the RSL3 and neratinib combination on growth inhibition in MDAMB415 cells with modest effects in MCF7 cells remains to be fully explained. The lower effectiveness of liproxstatin-1 in inhibiting lipid peroxidation induced by the RSL3 and neratinib combination in MCF7 compared to MDAMB415 cells may be a contributing factor.

### RSL3 and neratinib induces replication stress

**Fig. 4:**
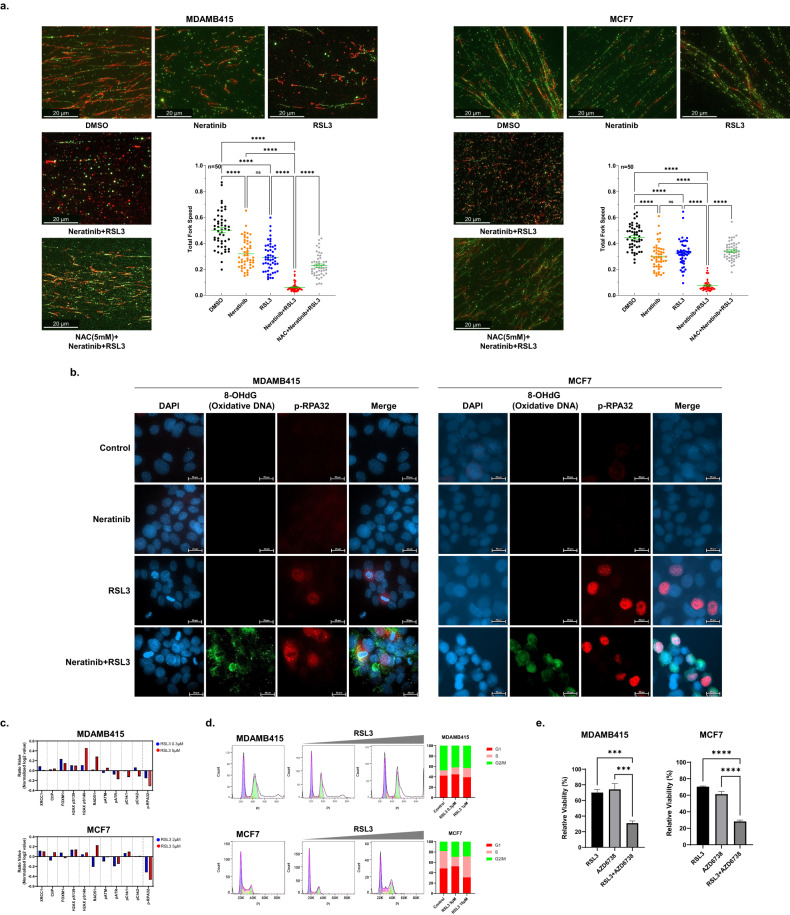
RSL3 and neratinib induce replication stress. **a** Cells were treated with the indicated inhibitors (MDAMB415: 1 µM for mono and combination therapy, MCF7: 10 µM for mono and combination therapy) for 24 h, and fork speed was assessed by DNA fiber assay (see Methods). Data are mean ± SEM. Scale bars: 20 μm. *p*-values were calculated with a one-way ANOVA test. ****p < 0.0001. **b** Cells were treated with the indicated inhibitors (MDAMB415: 1 µM for mono and combination therapy, MCF7: 10 µM for mono and combination therapy) for 24 h. Representative images of 8-OHdG/phospho-RPA32 immunofluorescence staining were shown. Scale bars: 20 μm. **c** Cells were treated with RSL3 (MDAMB415: 0.3, 5 μM, MCF7: 2, 5 μM) for 3 days. DNA damage-related protein levels are presented as the ratio of treated/control from RPPA data. **d** Cells were treated with RSL3 (MDAMB415: 0.3, 1 μM, MCF7: 5, 10 μM) for 1 day. Cell cycle progression was detected by staining of PI with flow cytometry. **e** Cell viability was assessed by presto blue assay with ATR inhibitor (AZD6738, 0.25 μM for MDAMB415 and 0.0625 μM for MCF7) and RSL3 (0.25 μM for MDAMB415 and 0.0625 μM for MCF7) for 6 days. *p*-values were calculated with a one-way ANOVA test. ***p < 0.001, ****p < 0.0001.

While it was clear that the combination of RSL3 and neratinib induced ferroptosis, inhibiting ferroptosis did not completely reverse the effects of the combination on cell viability. Therefore, we investigated additional mechanisms that could contribute to the growth inhibition and cell death induced by RSL3 and neratinib. As ROS can induce replication stress (RS), and RS, in turn, can induce ROS [27], we investigated whether the combination of neratinib and RSL3 induced ROS production and its downstream consequences and whether this could also induce RS. As indicated by DNA fiber, both neratinib and RSL3 induced a modest increase in RS, as indicated by a decrease in fork speed (Fig. 4a). However, the combination of neratinib and RSL3 induced a marked decrease in fork speed in both MDAMB415 and MCF7 cells consistent with marked induction of RS (Fig. 4a). Consistent with the RS being induced, at least in part by ROS, NAC partially reversed the effects of RSL3 and neratinib on RS (Fig. 4a). RS is associated with the accumulation of single-strand DNA, which is coated by pRPA32 [28]. As indicated in Fig. 4b, pRPA32 and 8OHdG (indicative of oxidative DNA damage that is induced by ROS and is associated with ferroptosis [29, 30]) were modestly increased by RSL3 and markedly increased by the combination of RSL3 and neratinib in both MDAMB415 and MCF7 cells (Fig. 4b), as well as in the LPA2/T33 syngeneic mouse model in vivo (Supplementary Fig. 4a). Moreover, the combination of cobimetinib with RSL3 increased oxidative DNA damage/replication stress (8-OHdG and phospho-RPA32) compared to cobimetinib or RSL3 treatment alone in MDAMB415 and MCF7 cell lines (Supplementary Fig. 4b). This suggests that the effects of neratinib are mediated, at least in part, through decreased RAS/MEK pathway signaling.

Interestingly, RSL3 alone was sufficient to induce a degree of RS. As multiple drugs have been proposed to induce or capitalize on therapy-induced RS [31], this finding suggested a potential additional therapeutic opportunity. As indicated by Fig. 4c, RPPA analysis supported the concept that RSL3 was sufficient to induce RS. This was further supported by the observation that RSL3 induced a dose-dependent increase in cells in the S phase of the cell cycle in MCF7 cells with a lesser increase in MDAMB415 cells (Fig. 4d). Consistent with these findings, inhibition of the RS detection and checkpoint inducer, Ataxia telangiectasia and rad3-related (ATR) using AZD6738, increased the growth inhibition induced by RSL3 in both the RSL3-resistant MDAMB415 and MCF7 cell lines (Fig. 4e).

## Discussion

The majority of luminal BC cell lines were highly sensitive to monotherapy with the GPX4 inhibitor RSL3. Interestingly, they exhibited much lower sensitivity to the xCT inhibitor erastin. This suggests that as GPX4 inhibitors enter the clinic, that luminal BC could be a potential therapeutic target for GPX4 inhibitors. However, a subset of luminal BC cell lines exhibited resistance to the effects of the RSL3 tool compound, providing an opportunity to explore mechanisms of RSL3 resistance, as well as an opportunity to identify combination therapies that might prevent the acquisition of resistance to drugs targeting GPX4 or reverse resistance once it occurs. Our studies demonstrated that in RSL3-resistant cell lines, RSL3 induced activation of the HER2 pathway that could represent an adaptive resistance mechanism to RSL3. Although the RSL3-resistant BC cell lines do not have amplified HER2, they do appear to express HER2 and demonstrate HER2 phosphorylation and signaling as assessed by RPPA, which represents a highly sensitive assay [32]. The expression of HER2 and HER2 activation in the luminal BC cell lines without HER2 amplification is consistent with the activity of trastuzumab-deruxtecan in a broad spectrum of BC including those without HER2 amplification.

The challenge in moving GPX4 inhibitors likely relates, at least in part, to difficulties in generating clinically relevant compounds. Further, ferroptosis is a critical component of normal cellular homeostasis. Indeed a number of papers have emphasized the challenge of identifying an adequate therapeutic index due to the role of ferroptosis in normal cells [33, 34]. This challenge may be resolved by the identification of biomarkers able to identify patients likely to demonstrate marked benefit to GPX4 inhibition either as monotherapy or combination therapy.

Adaptive resistance represents an important mechanism of therapy resistance that is therapeutically tractable [22]. Indeed, we were able to demonstrate that the irreversible HER2 inhibitor neratinib was synergistic with RSL3 in the endogenously RSL3-resistant cell lines, as well as in a cell line that was made resistant to RSL3 through culture with increasing drug concentrations consistent, thus confirming HER2 activation represents an adaptive resistance mechanism to the combination of RSL3 and neratinib. Interestingly, while additional irreversible HER2 inhibitors (afatinib and dacomitinib) recapitulated the synergy with RSL3 observed with neratinib, inhibitors targeting EGFR (gefitinib) or reversible (lapatinib, sapitinib, and tucatinib), HER2 inhibitors were not synergistic with RSL3. The mechanism that distinguishes irreversible from reversible HER2 inhibitors remains to be elucidated but it may be related to the prolonged inhibition of HER2 signaling induced by irreversible inhibitors. Importantly, in a transplantable syngeneic estrogen-dependent BC tumor model [24], the combination of RSL3 and neratinib was more effective than either drug alone and further was not toxic in the murine model providing support for the transition to clinical trials.

In the resistant cell lines, RSL3 not only had modest effects on growth inhibition but also on a number of processes involved in ferroptotic cell death such as the induction of ROS production, increased mitochondrial iron levels, mitochondrial damage, and lipid peroxidation. However, the combination of neratinib and RSL3 was highly effective at inducing these processes in the RSL3-resistant cell lines. Furthermore, inhibiting of ROS, GSH, and iron chelation partially reversed the activity of the neratinib and RSL3 combination. Additionally, the synergy observed with RSL3 was mimicked with other GPX4 inhibitors, as well as with GPX4 knockdown. Together this supported ferroptosis as the key process underlying the effects of the RSL3 and neratinib combination. In MDAMD415 cells, liproxstatin-1, as well as an additional lipid peroxidation inhibitor, markedly decreased the growth inhibition induced by the combination of RSL3 and neratinib. However, in MCF7 cells, liproxstatin-1 had only modest effects on the efficacy of the RSL3 and neratinib combination. Furthermore, liproxstatin-1 did not reverse the effects of high-dose RSL3 monotherapy activity in MCF7 cells consistent with liproxstatin-1 failing to efficiently inhibit lipid peroxidation in MCF7 cells. Interestingly, liproxstatin-1 did inhibit the effects of neratinib and RSL3 on lipid peroxidation, however, liproxstatin-1 appeared to be less efficient at blocking lipid peroxidation in MCF7 cells suggesting that residual lipid peroxidation could provide a potential explanation for the limited effects of liproxstatin-1 on growth inhibition by RSL3 and neratinib. Another potential mechanism for the differential response to liproxstatin-1 include the underlying genomic aberrations in the lines with for example MDAMB415 being *TP53* mutant and MCF7 *TP53* wild type and MCF7 but not MDAMB415 having a functional *PIK3CA* mutation. The relationship between p53 function and ferroptosis pathway activation is controversial. p53 has been reported to delay ferroptosis through a p21-dependent mechanism [35]. In contrast, MDM2 and MDMX, negative regulators of p53, have been reported to promote ferroptosis through the induction of PPARα activity [36].

In addition to ferroptosis, RSL3 induced RS and sensitivity to the ATR RS resistance mediator. RS has been demonstrated to increase ROS as well as be caused by ROS. Thus, it is possible that the two processes (RS and ferroptosis) are mechanistically linked [27].

Taken together, the preclinical data suggest that when GPX4 inhibitors become clinically available, luminal BC, which causes more deaths than all other forms of BC combined, should represent a high-quality target. The combination of a GPX4 inhibitor and an irreversible HER2 pathway inhibitor should be considered to reverse pre-existing or acquired resistance or potentially prevent or delay the emergence of resistance. An alternative approach might be the combination of a GPX4 inhibitor and an inhibitor of the ATR/CHK1/WEE1 pathway.

## Materials and methods

### Cell lines and cell culture

All cell lines were cultured in a humidified incubator at 37 °C 5% CO_2_. The cell lines obtained from the ATCC (HCC202, MDAMB175VII, ZR75-30, CAMA1, BT474, T47D, MDAMB134VI, MDAMB453, MCF7, MDAMB415, ZR75-1, MCF10A, HCC1937, HCC1806, MDAMB231, and MDAMB436) and DSMZ (EFM19) were authenticated by fingerprint in the MD Anderson core or by ATCC, and tested to be free of mycoplasma contamination (ATCC, 30-1012 K). The Cells were cultured according to information provided by ATCC.

### Chemicals and drugs

RSL3 (S8155), neratinib (S2150), lapatinib (S2111), gefitinib (S1025), ML210 (S0788), afatinib (S1011), dacomitinib (S2727), sapitinib (S2192), Z-VAD-FMK (S7023), necrostatin-1 (S8037), 3-Methyladenine (3-MA, S2767), tucatinib (S8362), liproxstatin-1 (S7699), erastin (S7242), deferoxamine mesylate (DFO, S5742), deferiprone (S4067), ferrostatin-1 (S7243), cobimetinib (S8041), Trastuzumab (A2007) and AZD6738 (S7693) were obtained from Selleck Chemicals. L-glutathione (G6013) and N-Acetyl-l-cysteine (A9165) were purchased from Sigma-Aldrich. T-DM1 (HY-P9921) was obtained from MedChemExpress.

### shRNA lentivirus infection

Lentiviral constructs of GPX4#4 (TRCN0000046251), GPX4#5 (TRCN0000046252), and shRNA control (SHC001) were obtained from Sigma-Aldrich. Lentivirus was generated in the LentiX-293T transfected with packaging plasmids (psPAX2 and pMD2.G). Infected cells were selected with puromycin. Transfection efficiency was determined by qRT-PCR method [37] using TaqMan-assay probe GPX4 (Thermo Fisher Scientific, Hs00989766_g1) and normalized with ACTB (Thermo Fisher Scientific, Hs99999903_m1).

### Cell viability assays

Cells were treated with the indicated inhibitors various doses were applied with a 2-fold serial dilution for 72 h, and cell viability was measured using the PrestoBlue reagent (Thermo Fisher Scientific, A13261). Viability curves and IC50 values were generated by GraphPad software (IC50 values in Supplementary Table). Synergistic effects of combination treatments were calculated with CalcuSyn software using the Chou-Talalay equation definitions of additive effect (CI = 1), synergism (CI < 1), and antagonism (CI > 1) [38]. The graphs represent the mean and SD from triplicate samples, and the IC50 tables show the mean with SEM from three independent experiments. As the treatment did not reach the IC50 value at the highest concentration (10 μM) assessed, we marked 10 μM as the highest concentration for comparison with other inhibitors.

### RPPA

Cells were treated for 3 days with neratinib, RSL3, or a combination. Protein analysis was performed at the MD Anderson RPPA core lab. Detailed information on the procedures and antibodies used can be found on the website, https://www.mdanderson.org/research/research-resources/core-facilities/functional-proteomics-rppa-core.html.

### Cell cycle and 7AAD-positive staining

To detect the effect of therapy on cell cycle progression, cells were fixed with ice-cold 70% ethanol for at least 2 h, washed with PBS, and incubated with PI staining solution (BD, 556463). The cell cycle was analyzed using a Fortessa Flow Cytometer (BD Biosciences). For 7AAD staining, cells were collected with trypsinization, and washed with PBS, stained with 7AAD (Biolegend, 640922), and analyzed on a Fortessa Flow Cytometry. Results are from three independent experiments performed.

### MitoSOX levels

Cells were treated with the indicated inhibitors and then 5 μM MitoSOX (Thermo Fisher Scientific, M36008) was added for 10 min at 37 °C in a dark incubator. Cells were collected and red fluorescent intensity values indicating mitochondrial superoxide levels were detected using a Fortessa Flow Cytometer (BD Biosciences). Results are from three independent experiments performed.

### Lipid peroxidation, mitochondrial membrane potential, and mitochondrial iron

To analyze lipid peroxidation, cells were incubated with 10 μM BODIPY 581/591 C11 (Thermo Fisher Scientific, C10445) for 30 min at 37 °C. Fluorescent signals were detected using microscopy (EVOS FLoid Imaging System, Thermo Fisher Scientific) with lipid peroxidation being assessed by a shift from red 590 nm to green 510 nm representing lipid peroxidation. Mitochondrial membrane potential was assessed by staining the cells with a JC-1 MitoMP detection kit (Dojindo, MT09-10) for 30 min at 37 °C, followed by analysis using microscopy (EVOS FLoid Imaging System). Changes in fluorescence from green (530 nm-low membrane potential) to red (590 nm-high membrane potential) were recorded. For detection of mitochondrial iron, cells were stained using Mito-FerroGreen (Dojindo, M489-10) and Mitotracker Red FM (Thermo Fisher, M22425) for 30 min at 37 °C in serum-free media, and cells were washed with serum-free media. Serum-free medium containing inhibitors was added for 3 h and detected with microscopy (EVOS FLoid Imaging System). Images were randomly taken from three different regions of interest (ROI) and are representative of three independent experiments.

### GSH

Total GSH in cell lysates was measured using a GSH assay kit (Sigma, CS0260) according to the manufacturer’s instructions. The graphs represent the mean and SD from triplicate tests conducted in three independent experiments.

### Animal study

LPA2/T33 tumor tissue from the LPA2/T33 transgenic syngeneic mouse model [24] was transplanted into the mammary fat pad of FVB/NJ mice (JAX Stock# 001800, female, 4–6 weeks old). When tumors were around 100 mm^3^ volume (after 20 days), mice were randomized into 4 groups. The group received either vehicle (DMSO/PEG300/Tween80 for neratinib, DMSO/corn oil for RSL3), neratinib (3 mg/kg, every day, oral gavage), or RSL3 (30 mg/kg, twice a week, i.p.). Animal protocols were approved by the Institutional Animal Care and Use Committee (IACUC, TR01_IP00002062). Mice were sacrificed when tumors reached 2 cm^3^. Tumor size and mouse weight were measured every 3–4 days. Tumor volume was calculated by 1/2 (length x width^2^).

### IncuCyte monitoring

Cell proliferation and death were monitored every 3 h using an IncuCyte system (Sartorius, IncuCyte S3). Proliferation was assessed by calculating the confluence value, and cell death was evaluated based on the red fluorescence (µm^2^/Image) of Ethidium homodimer-1 (EthD1, Thermo Fisher Scientific) staining. Results are derived from three independent experiments.

### DNA fiber assay

Cells were treated with the indicated drugs prior to fiber analysis [39]. The cells were labeled with 20 μM CldU (Sigma-Aldrich, I7125) for 20 min, washed twice with PBS, and labeled with 200 μM IdU (Sigma-Aldrich, C6891) for 60 min at 37 °C. Graphs represent the mean with SEM from fifty different DNA strands. Results are derived from three independent experiments.

### Immunofluorescence staining

Cells were treated with the indicated inhibitors and fixed with 4% paraformaldehyde for 15 min at RT. Fixed cells were washed with PBS and blocked with 5% normal goat serum/0.3% Triton X100/PBS for 1 h. FFPE slides (at least three FFPE slides in each group) were obtained from the LPA2/T33 syngeneic mouse mode. Slides were deparaffinized, subjected to graded rehydration, antigen retrieval (citrate buffer, pH 6.0), and blocked with 5% normal goat serum/PBS [40]. Primary antibodies (8-OHdG (Abcam, ab62623), p-RPA32 (Bethyl, A300-245A)) were incubated overnight at 4 °C, followed by incubation with secondary antibodies for 1 h in the cell. Slides were mounted with ProLong Gold antifade with DAPI (Thermo Fisher Scientific, S36938). Images were captured using a ZEISS Apotome 3 microscope. Results are based on three independent experiments.

### Gene-set enrichment analysis (GSEA)

RNAseq data were downloaded from the DepMap site (https://depmap.org/portal/download/all/) and gene expression was analyzed using a Ferroptosis gene-set comparing RSL3-sensitive vs. -resistant cells.

### Generation of RSL3-resistant cell line

To generate a RSL3-resistant cell line, RSL3-sensitive BT474 cells were incubated with gradually increasing doses of RSL3 to a final dose of 200 nM. RSL3 was removed for 2 weeks to identify “recovering cells”.

### GPX4, SLC7A11 and SLC3A2 expression

For BC cell line dataset analysis, baseline RNAseq data were downloaded from https://depmap.org/portal/download/all/. Subtypes were determined as described in reference [41]. For TCGA dataset analysis, Illumina RNASeq BRCA data were retrieved from TCGA using the TCGAbiolinks package. TCGA data were filtered down to the desired gene groups using the tidyverse package. Statistics were assessed with ANOVA tests across individual genes, subtypes, and counts. Significance was confirmed with a Tukey test. Individual visualizations for each gene and their corresponding subtypes were created using the ggplot2 package.

### SLC7A11 and SLC3A2 protein detection

To detect SLC7A11 and SLC3A2 protein expression levels, cells were suspended in cell staining buffer and stained with SLC3A2 FITC antibody (Thermo Fisher Scientific, 11-0982-42), SLC7A11 (Cell Signaling Technology, 12691) or the corresponding isotype control antibody (Thermo Fisher Scientific, 11-4714-42; Cell Signaling Technology, 2975S) for 20 min at RT. For SLC7A11, this was followed by secondary antibody incubation for 20 min. Cells were washed with cell staining buffer and analyzed using a Fortessa Flow Cytometer. Experiments were performed in triplicate.

### Western blot analysis

The cells were lysed using RIPA lysis buffer (Sigma-Aldrich, R0278) supplemented with a proteinase inhibitor (Roche, 04693124001) and a phosphatase inhibitor (Roche, 046906837001). The protein concentration in the supernatant was determined using a BCA protein assay (Bio-Rad) [40]. Antibodies against phosphorylated MAPK (T202/Y204) (Cell Signaling Technology, 4377), MAPK (Cell Signaling Technology, 4695), GPX4 (Cell Signaling Technology, 52455S), and β-Tubulin (Santa Cruz, sc-47778) were used. The results of are representative three independent Western blot experiments performed in triplicate.

### Cystine uptake measurement

Cystine uptake was measured using the cystine uptake assay kit (Dojindo, UP05-12). Cells were washed three times with HBSS (Thermo Fisher Scientific, 14025092) and then incubated in cysteine-free DMEM (Thermo Fisher Scientific, 21013024) for 5 min at 37 °C. Subsequently, cells were treated with neratinib, RSL3, erastin, or a combination of neratinib and RSL3 in 200 μl of pre-warmed cystine uptake solution for 30 min. After washing with HBSS, cells were incubated with methanol and fluorescence solution for 30 min at 37 °C. The fluorescence intensity was measured using a fluorescence plate reader (Ex/Em = 490/535 nm). The results represent three independent experiments performed in triplicate.

### Cancer therapeutics response portal v2 (CTRPv2) dataset

AUC of RSL3, ML210, and ML162 in luminal BC set was downloaded from the NCI CTD^2^ Data Portal at:

ftp://caftpd.nci.nih.gov/pub/OCG-DCC/CTD2/Broad/CTRPv2.1_2016_pub_NatChemBiol_12_109/).

### Statistical analysis

Comparison between the two groups was analyzed using an unpaired Student’s *t*-test. One-way or two-way analysis of variance (ANOVA) with Tukey’s multiple comparisons test was used to compare among groups. The data are shown as the mean ± standard deviation (SD) or mean ± standard error of the mean (SEM) as indicated. All statistical tests were conducted using Prism software (GraphPad).

## Supplementary information

Supplementary Figure legend

Supplementary Figure1

Supplementary Figure2

Supplementary Figure3

Supplementary Figure4

Supplementary Table

Original Data File

Original Data File

Reproducibility checklist

## Data Availability

All data generated or analyzed during this study are available from the corresponding author on reasonable request.
