# Supplementary material for: Irreversible HER2 inhibitors overcome resistance to the RSL3 ferroptosis inducer in non-HER2 amplified luminal breast cancer
Source: Cell Death Dis. 2023 Aug 18;14(8):532. doi: 10.1038/s41419-023-06042-1 (PMC10439209; doi:10.1038/s41419-023-06042-1)
Supplement: Supplementary file 1 — Supplementary Figure legend [file 41419_2023_6042_MOESM1_ESM.pdf]

Supplementary Figure.

Supplementary Fig. 1. Sensitivity to ferroptosis in luminal BC cell lines.

(a) Correlation of RSL3 IC50 values (from Fig. 1a) with AUC of RSL3, ML210 or ML162 from CTRPv2 in luminal BC cell lines. (b-c) Proteins were extracted from four luminal breast cancer cell lines (MDAMB415, ZR75-1, MCF7 and CAMA1), four basal breast cancer cell lines (MDAMB436, MDAMB231, HCC1937 and HCC1806), and one normal breast epithelial cell line (MCF10A). Protein expression levels of GPX4 were determined by Western blot (b), while SLC7A11 and SLC3A2 levels were quantified by FACS analysis (c). The results are presented as a scatter dot plot with the line indicating the median value. (d) GSEA analysis of RSL3 sensitivity with a transcriptome levels using WIKIPATHWAYS-ferroptosis pathway for RSL3 sensitive (left side) vs resistant cell lines (right side).

Supplementary Fig. 2. Irreversible HER2 inhibitors are synergistic with RSL3 in RSL3 resistant cell lines.

(a) Irreversible (afatinib or dacomitinib) or reversible HER2 inhibitors (sapitinib or tucatinib) were incubated with and without RSL3 (0-10μM) for 3 days in RSL3 resistant cell lines. Viability was assessed with presto blue. CI values of combination treatment were calculated by the Chou-Talalay equation in CalcuSyn. (b) Cells were treated with erastin (0-10μM) or neratinib (0-10μM) for 3 days and cell viability was assessed by presto blue. (c) MCF7 cells were treated with 0-10μg/ml of Trastuzumab or T-DM1 in the presence or absence of RSL3 (0-10μM) for a period of 3 days. Cell viability was assessed using presto blue. (d) MCF7 cells were seeded in a 96-well plate, and 200 μl of prewarmed CA uptake solution containing neratinib (10μM), RSL3 (10μM), combination (10μM, each), or erastin (100μM) was added for 30 min incubation. The data presented represents the mean with standard deviation in triplicate, normalized by protein concentration. (e) qPCR assay of knockdown efficacy in cells from Fig. 2b. (f) Cells were treated with ML210 (GPX4 inhibitor, 0-10μM) and neratinib (0-10μM) for 3 days and cell viability was assessed with presto blue. (g) MDAMB415 and MCF7 cells were treated with neratinib, RSL3, or combination

(MDAMB415: 0.3, 1, 5μM, MCF7: 5, 10μM for single and combination) for 3 days and 7AAD positive cells were assessed by FACS analysis. (h) Individual tumor weight measurements were taken after dissection on day 37 (for the control group) or day 45 (for the treatment group). Tumor weights are presented as mean ± SD. p values were calculated with Two-way ANOVA with multiple comparisons test. *p<0.05 compared with control. (i) Mouse body weight of mice in Fig. 2g presented as mean±SD.

Supplementary Fig. 3. RSL3 and neratinib induce ferroptosis in RSL3-resistant cells.

(a) ZR75-1 cells were treated with neratinib and RSL3 (N+R, 1.25μM each) with the indicated doses of GSH (1mM) or liproxstatin-1 (5μM) for 3 days and viability assessed with presto blue. (b) Mitochondrial membrane potential was measured with JC-1 staining in neratinib and RSL3 treated cells (MDAMB415: 0.3, 1, 5μM, MCF7: 5, 10μM) after 1 day. Scale bars: 200 μm. (c) Cell growth was assessed by IncuCyte systems every 3 h for 6 days of cells treated with neratinib and RSL3 (MDAMB415: 1μM, MCF7: 10μM) with and without ferroptosis inhibitors (liproxstatin-1: 5μM, ferrostatin-1: 10μM) or GSH (1mM). (d) RSL3 resistant cells were treated with different doses of RSL3 or neratinib with and without liproxstatin-1 5μM for 3 days. Cell growth was assessed using an IncuCyte every 3 h for 6 days. (e) MCF7 cells were seeded in a 24 well plate, and treated with cobimetinib (2μM), RSL3 (2μM), or combination (2μM, each) for 3 h. Lipid peroxidation was detected with BODIPY 581/591 C11 staining (oxidized, green). Scale bar: 200 μm. Imaris software was used for cell segmentation and quantification of green and red intensity per cell, which was then normalized with cell number and represented as a relative amount. (f) Cells were treated with afatinib (10μM), RSL3 (10μM), combination (10μM, each), or dacomitinib (10μM) for 3 h. Mitochondrial iron was detected with Mito-FerroGreen with costaining with Mitotraker to identify mitochondria. Scale bars: 100 μm. (g) Cells were treated with the afatinib (10μM), RSL3 (10μM), combination (10μM, each), or dacomitinib (10μM) for 3 h. Mitochondrial ROS was detected by MitoSOX staining with flow cytometry analysis. (h) MCF7 cells were treated with afatinib (10μM), RSL3 (10μM), combination (10μM, each), or dacomitinib (10μM) for 3 h. Cells were incubated with 10μM BODIPY 581/591 C11 for 30 min at 37°C.

Fluorescent signals were detected with microscopy. Results are representative of three independent experiments. Scale bar: 100 µm.

Supplementary Fig. 4. The combination treatment of RSL3 with either neratinib or cobimetinib induces replication stress accompanied by oxidative DNA damage.

(a) Oxidative DNA damage and replication stress markers were stained with 8-OHdG (green) and phospho RPA32 (purple) in the LPA2/T33 syngeneic mouse model. Scale bars: 10 µm. (b) Cells were treated with cobimetinib (2µM), RSL3 (2µM), or combination (2µM, each) for 24 h. DNA oxidative stress and replication stress were identified with 8-OHdG (green) and phospho-RPA32 (purple) respectively. Scale bars: 20 µm.
