# Supplementary figures and images for: Irreversible HER2 inhibitors overcome resistance to the RSL3 ferroptosis inducer in non-HER2 amplified luminal breast cancer
Source: Cell Death Dis. 2023 Aug 18;14(8):532. doi: 10.1038/s41419-023-06042-1 (PMC10439209; doi:10.1038/s41419-023-06042-1)

Supplementary Fig. 1

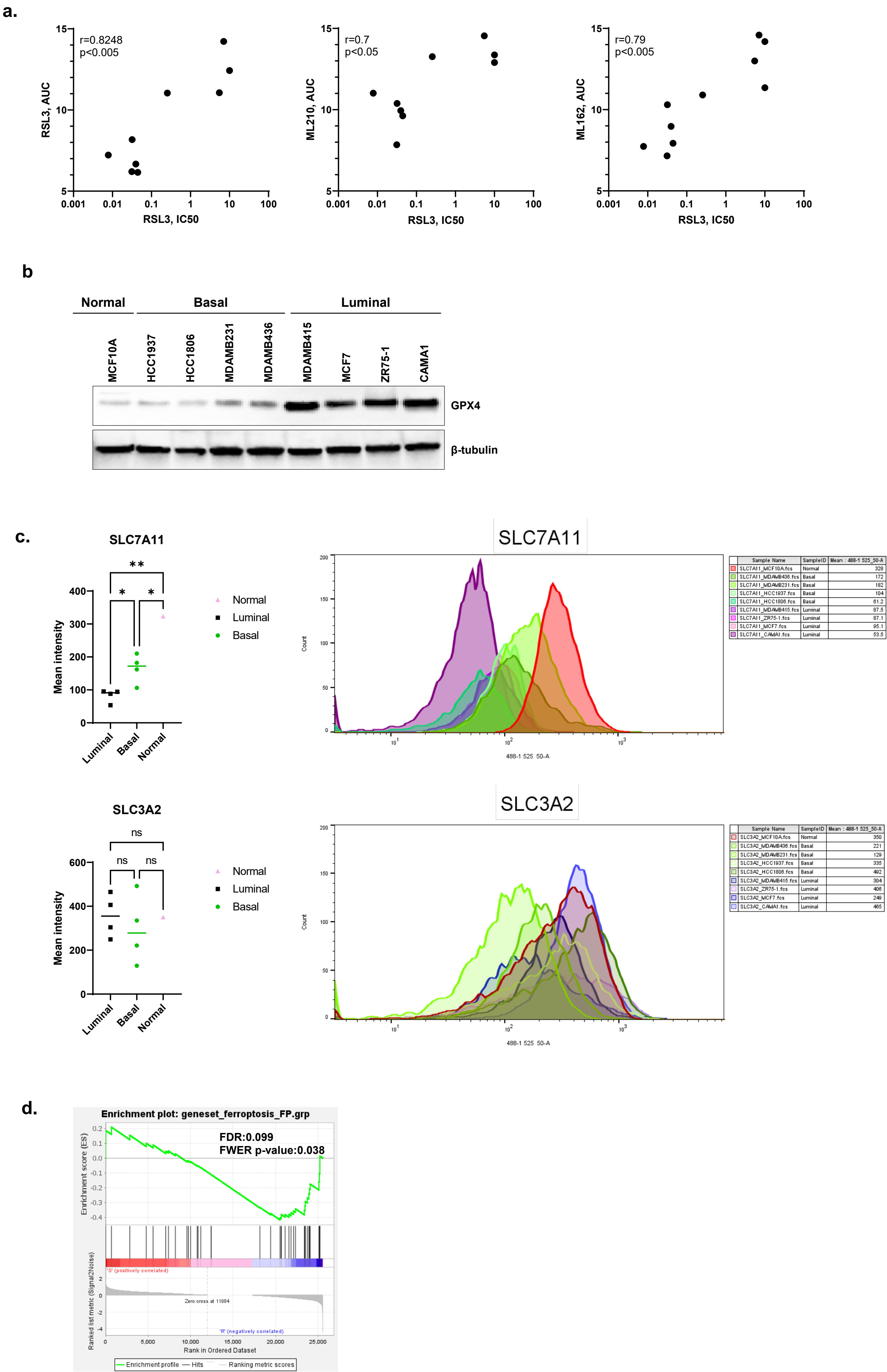

Supplement: Supplementary file 2 — Supplementary Figure1 [file 41419_2023_6042_MOESM2_ESM.pdf]

Supplementary Fig. 2

a.

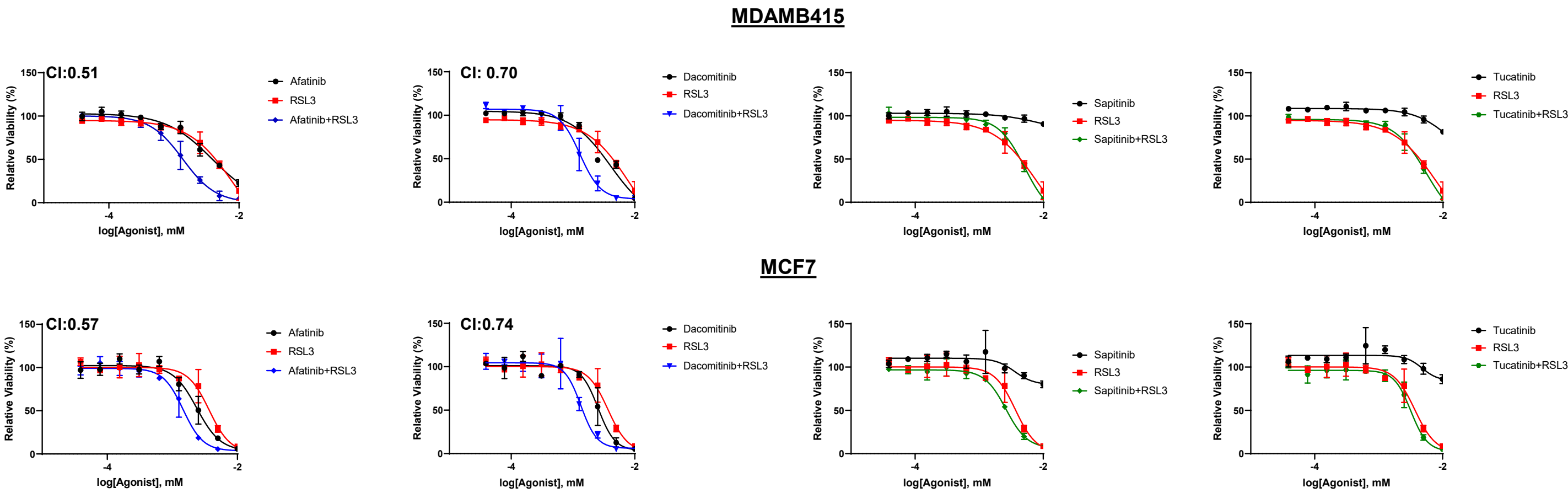

b.

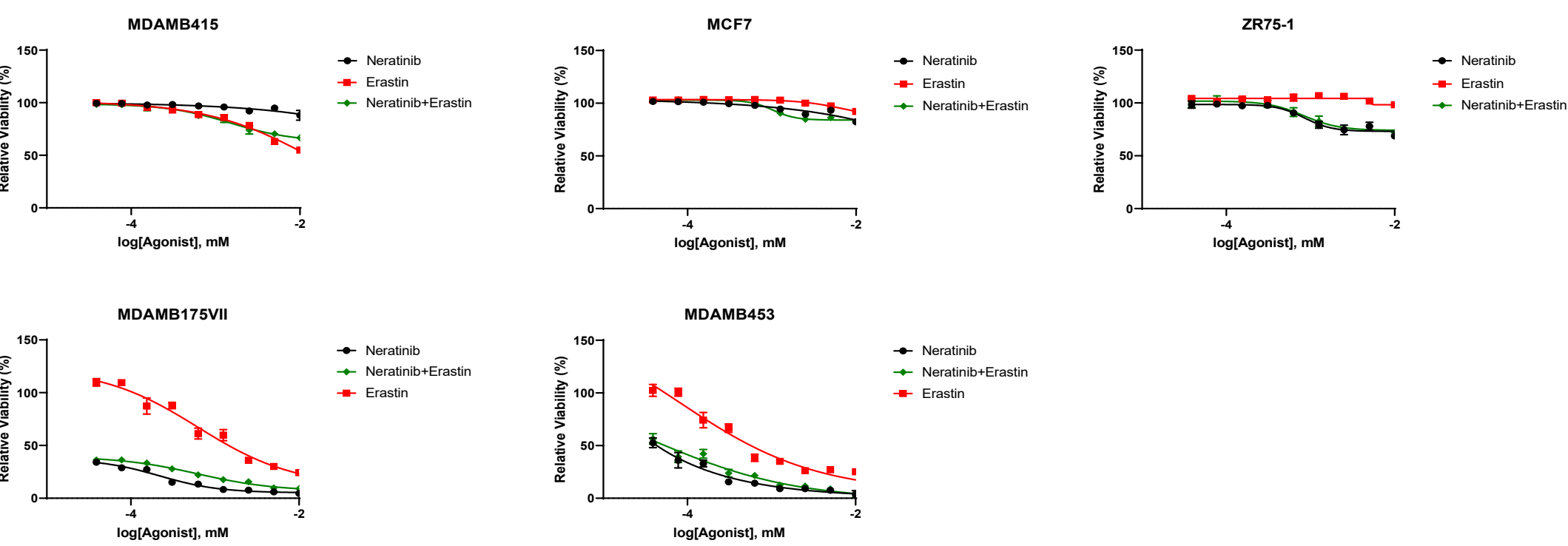

c.

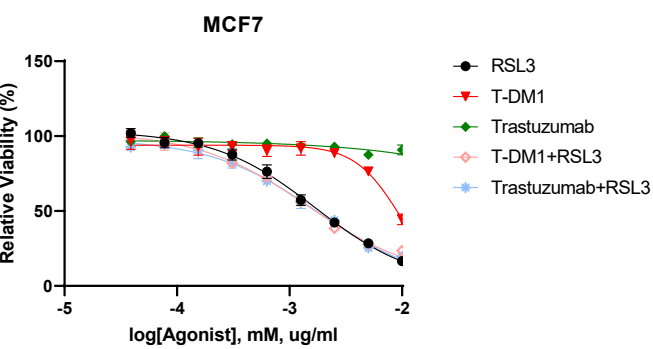

d.

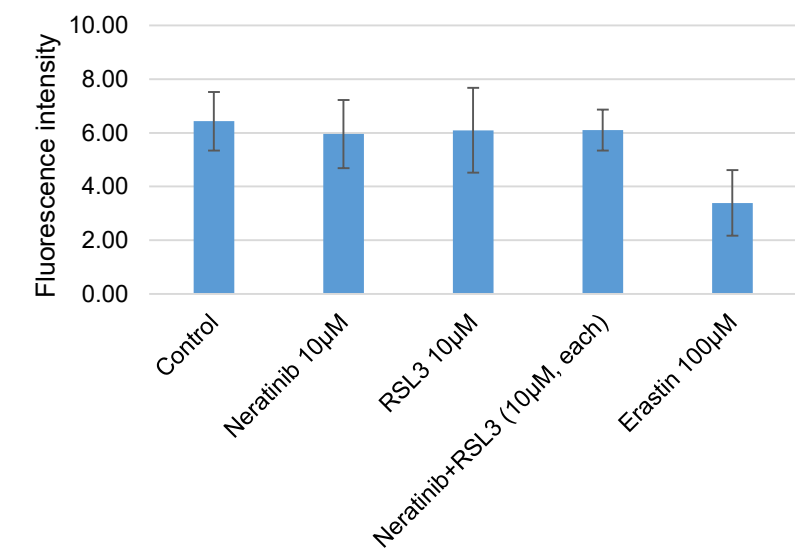

e.

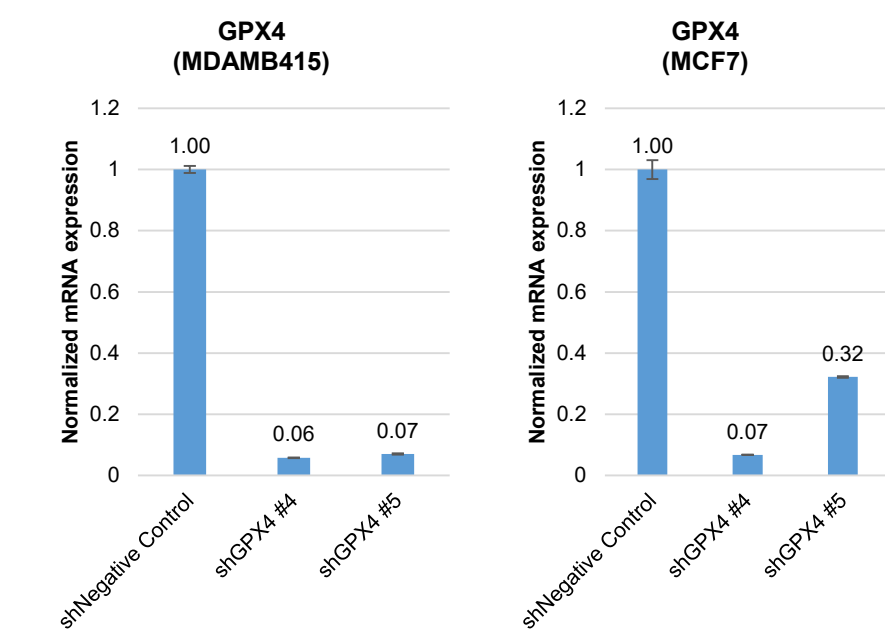

f.

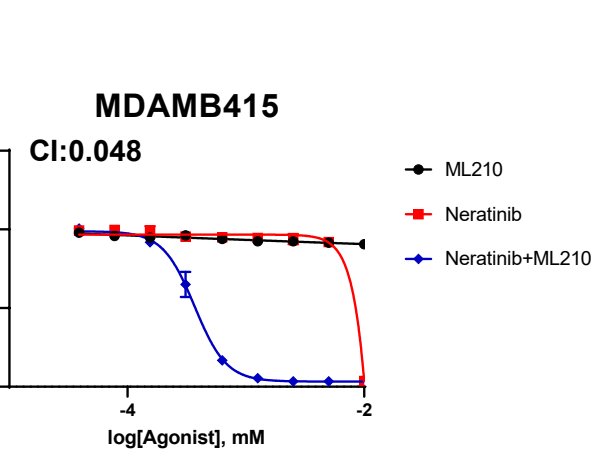

g.

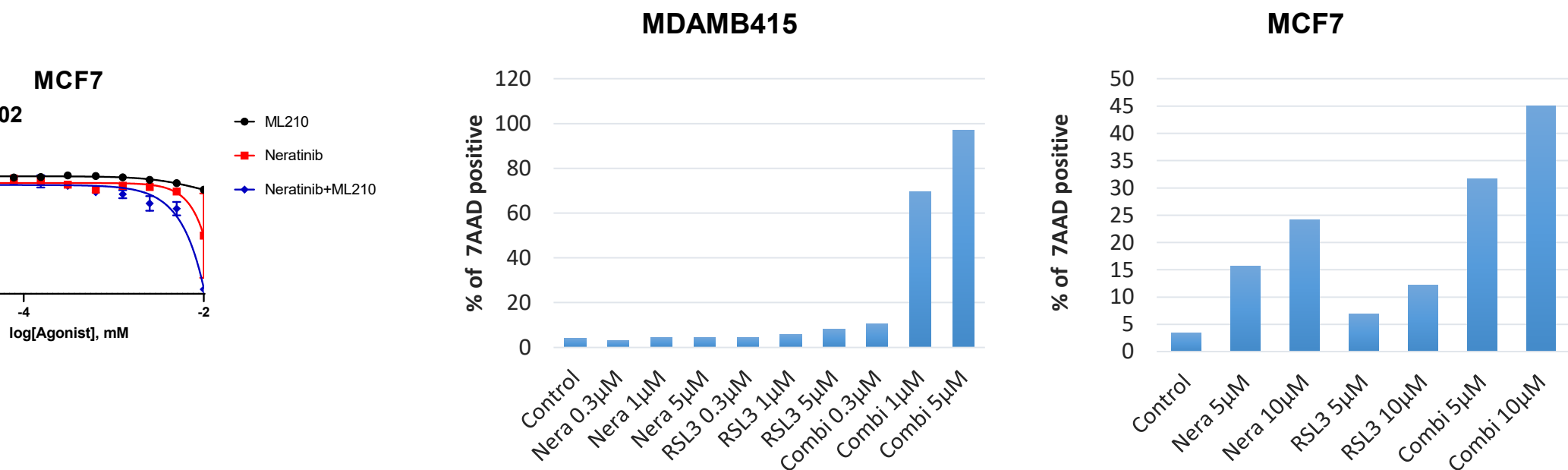

h.

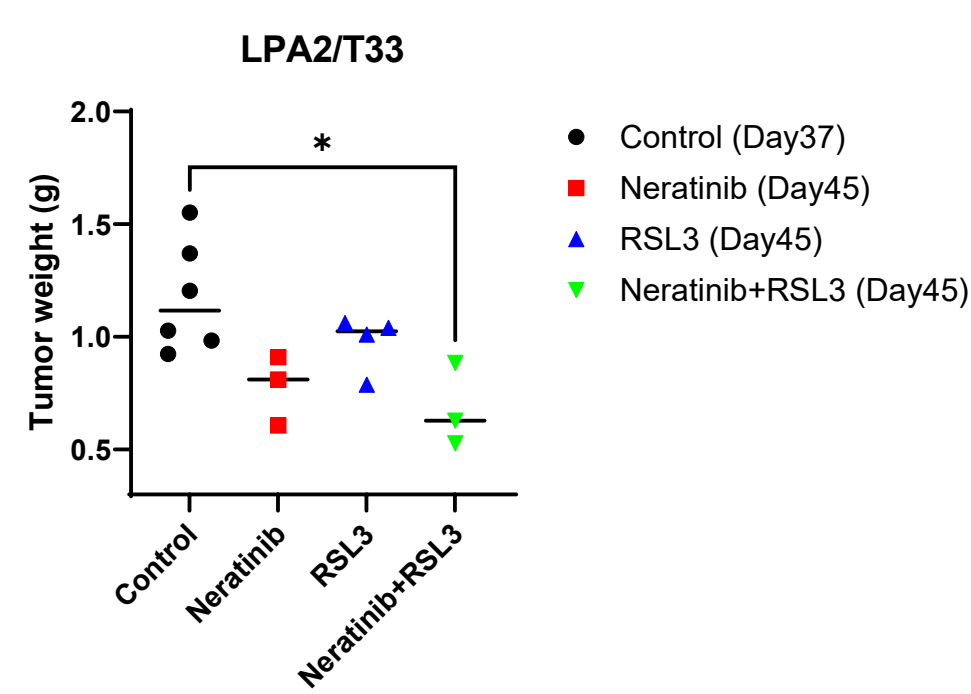

i.

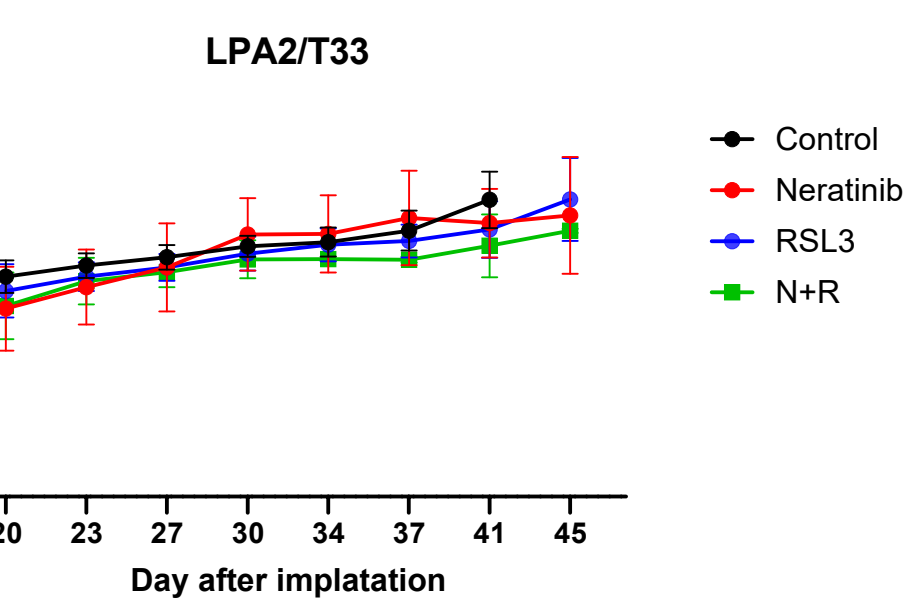

Supplement: Supplementary file 3 — Supplementary Figure2 [file 41419_2023_6042_MOESM3_ESM.pdf]

Supplementary Fig. 3

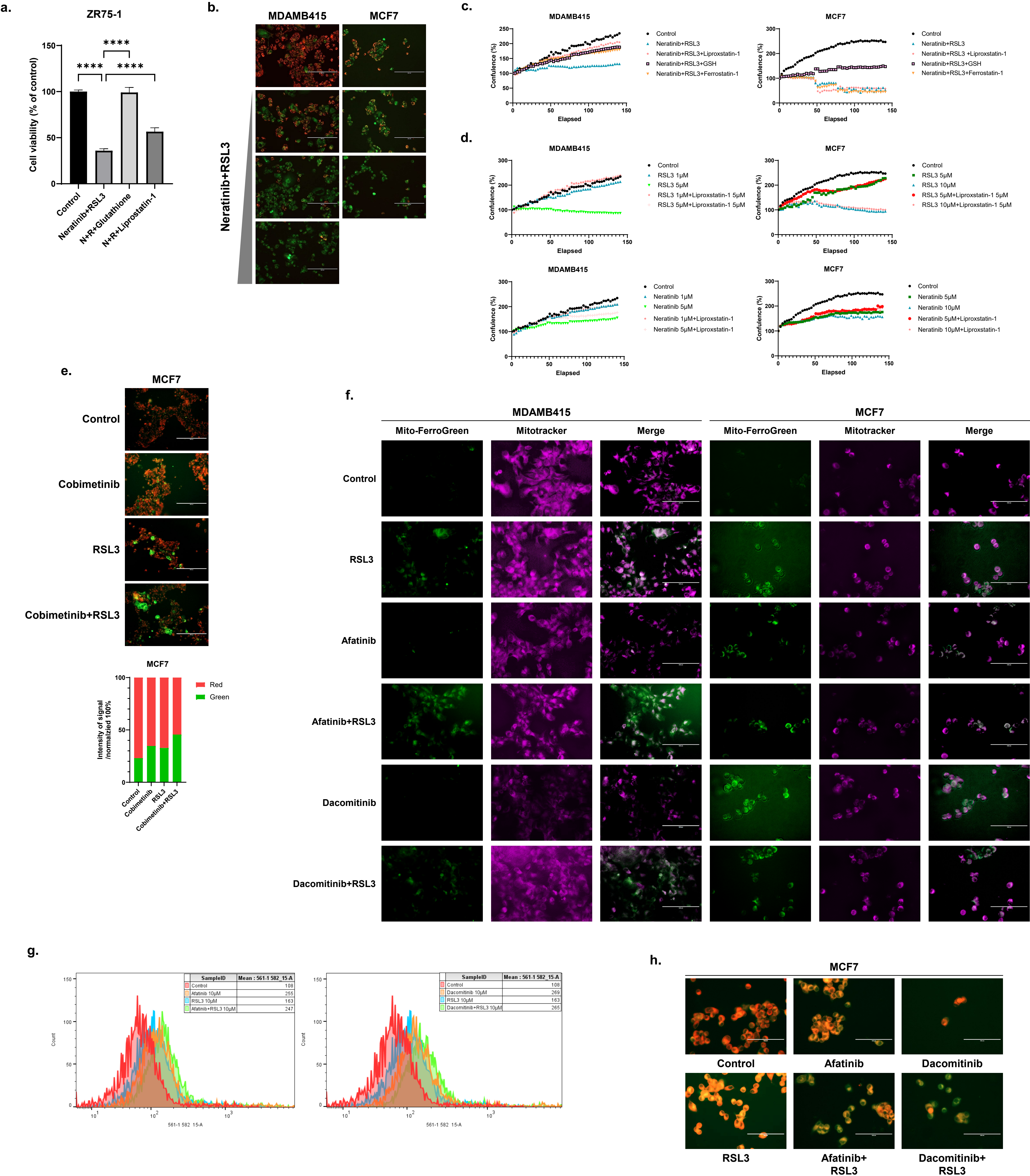

Supplement: Supplementary file 4 — Supplementary Figure3 [file 41419_2023_6042_MOESM4_ESM.pdf]

Supplementary Fig. 4

a.

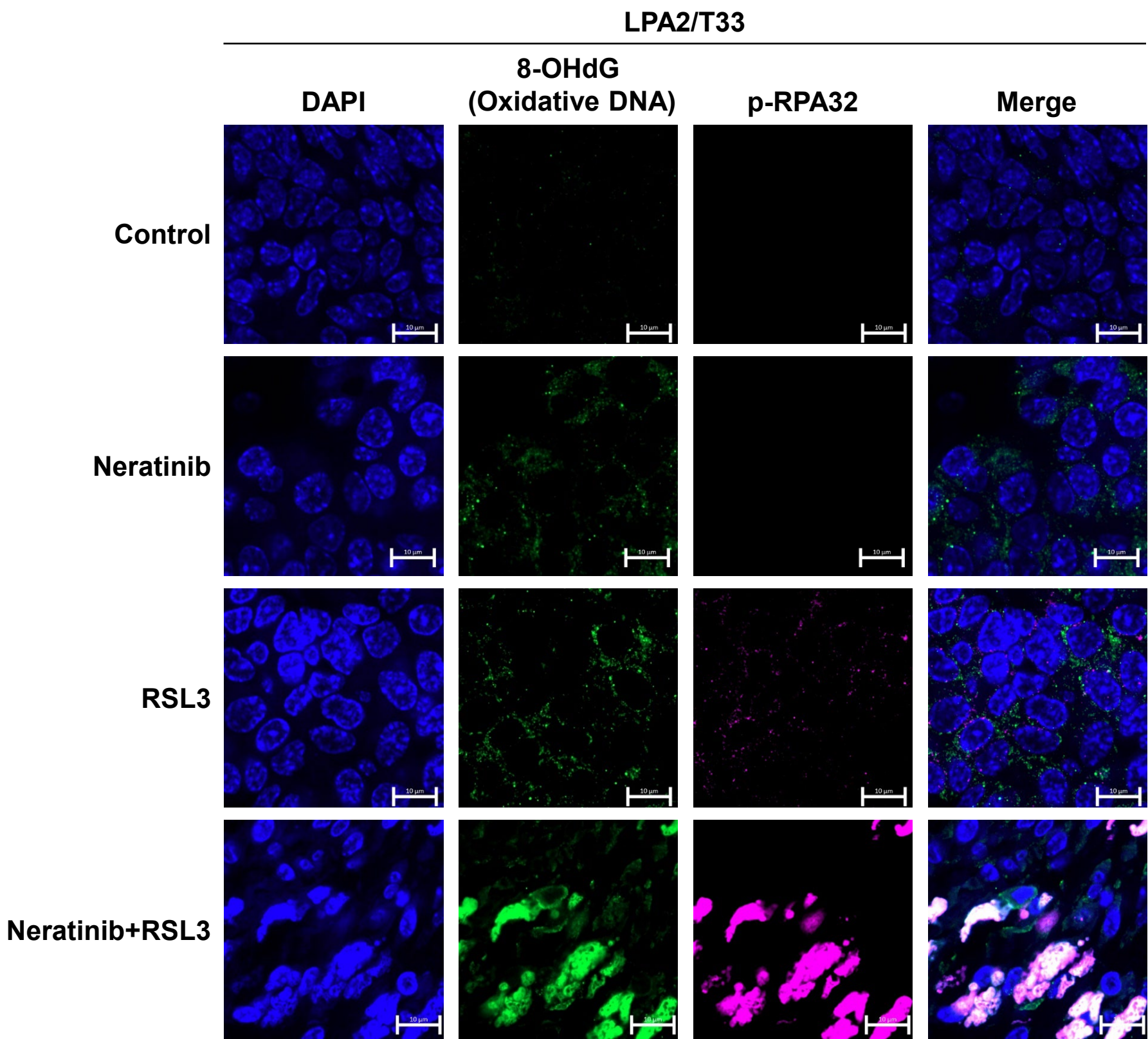

b.

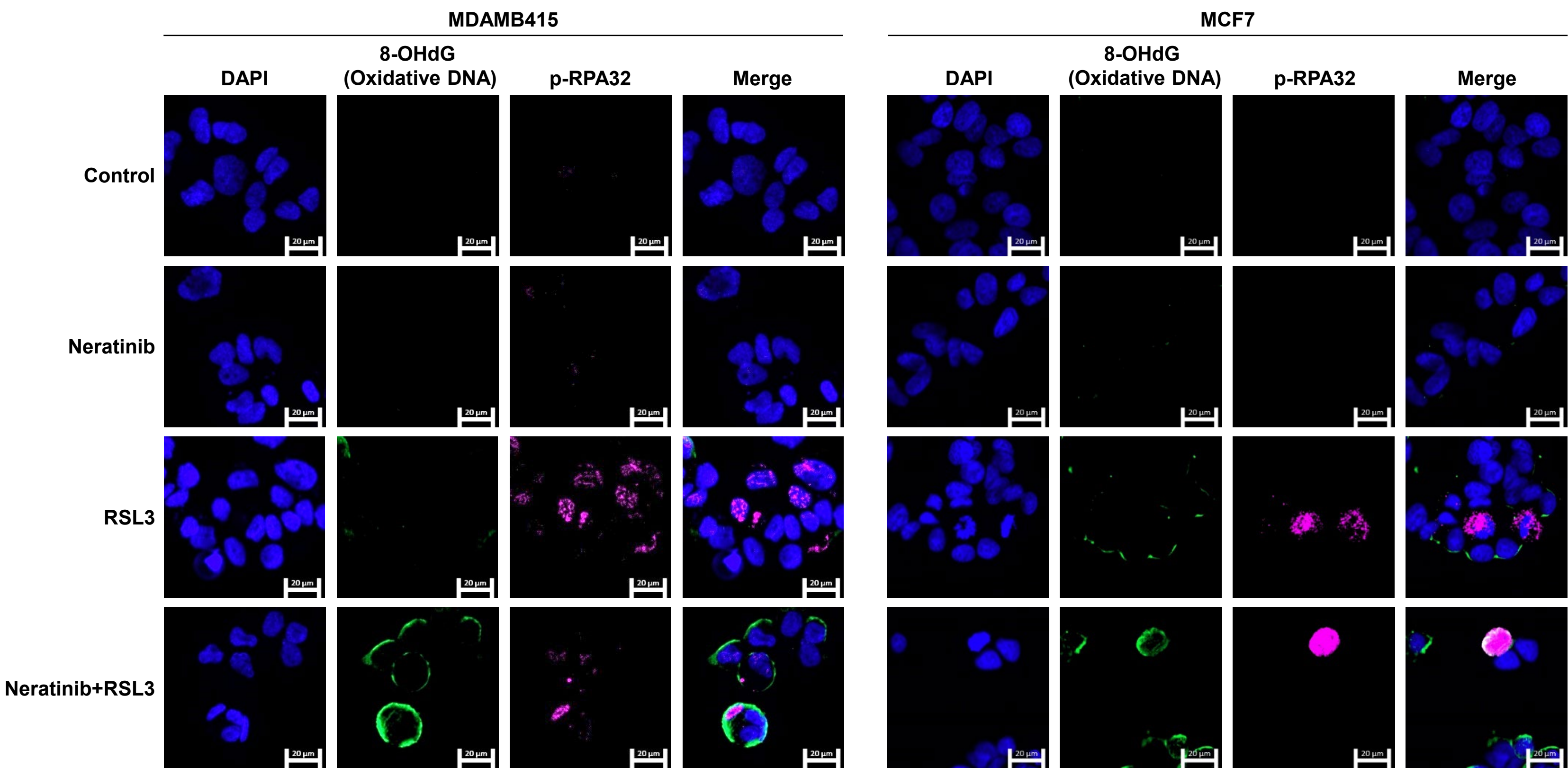

Supplement: Supplementary file 5 — Supplementary Figure4 [file 41419_2023_6042_MOESM5_ESM.pdf]

Fig. 2

d.

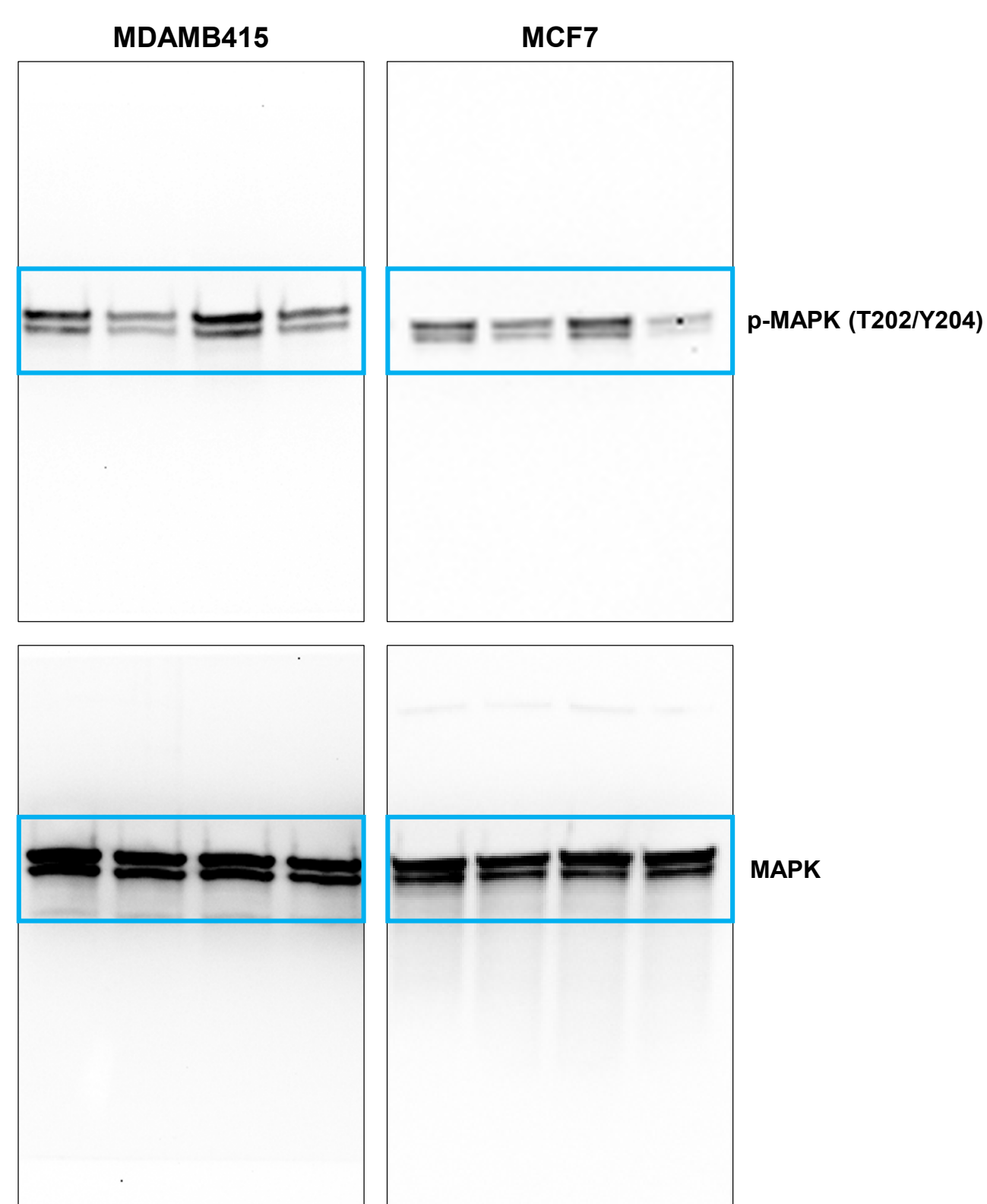

Supplement: Supplementary file 7 — Original Data File [file 41419_2023_6042_MOESM7_ESM.pdf]

Supplementary Fig. 1

**b**

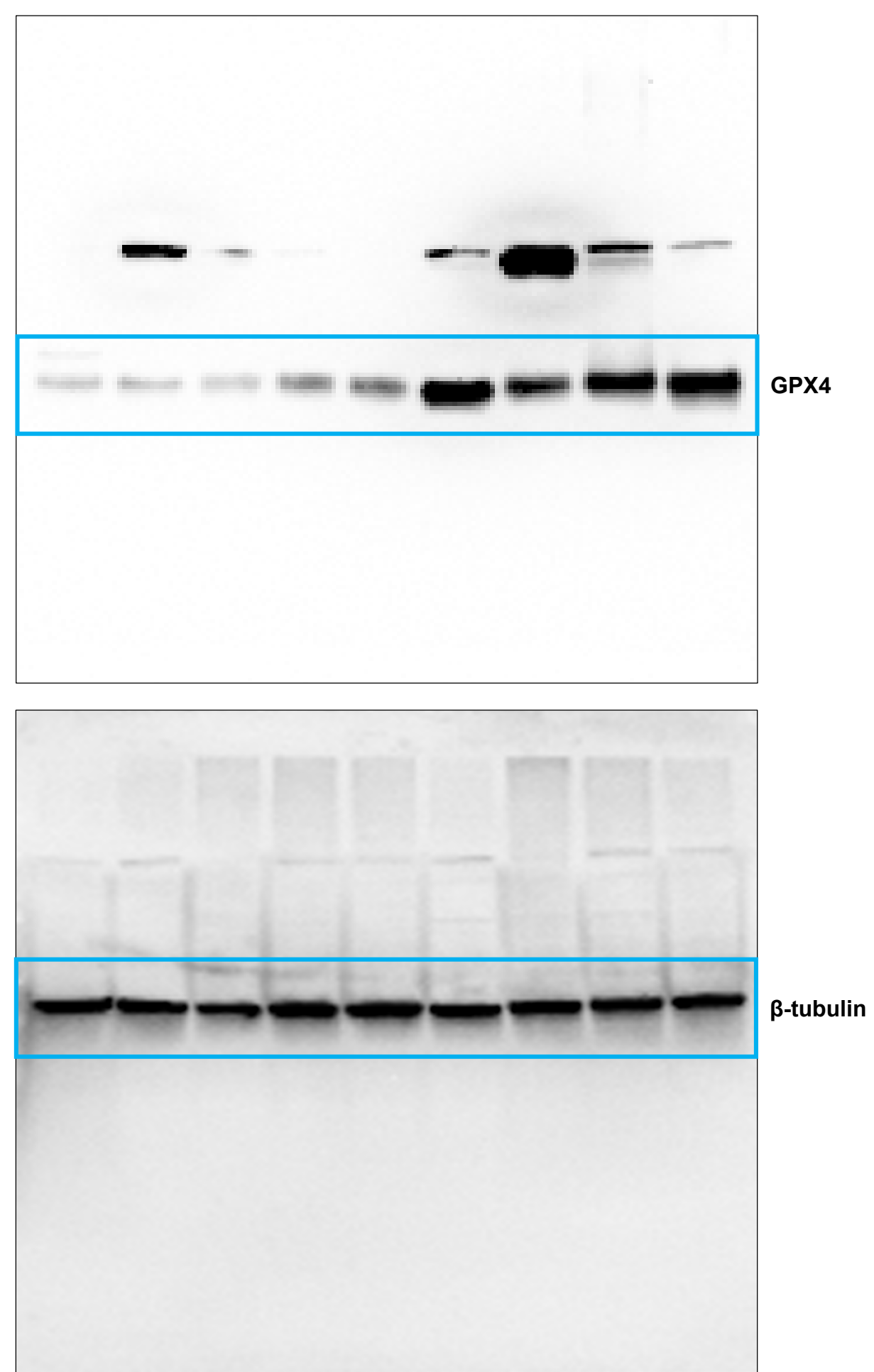

Supplement: Supplementary file 8 — Original Data File [file 41419_2023_6042_MOESM8_ESM.pdf]
