# Supplementary material for: Irreversible HER2 inhibitors overcome resistance to the RSL3 ferroptosis inducer in non-HER2 amplified luminal breast cancer
Source: Cell Death Dis. 2023 Aug 18;14(8):532. doi: 10.1038/s41419-023-06042-1 (PMC10439209; doi:10.1038/s41419-023-06042-1)
Supplement: Supplementary file 6 — Supplementary Table [file 41419_2023_6042_MOESM6_ESM.docx]

Table S1. RSL3 IC50 values were calculated from Fig. 1a. As erastin did not reach the IC50 values at the highest concentration (10µM) used in our study, we only included the IC50s table for RSL3.

| **Cell line** | **IC50 (Mean, µM)** | **SEM** |
| --- | --- | --- |
| MDAMB134VI | 0.071 | 0.003 |
| MDAMB453 | 0.391 | 0.063 |
| CAMA1 | 0.036 | 0.002 |
| MDAMB175VII | 0.221 | 0.102 |
| T47D | 0.040 | 0.004 |
| EFM19 | 0.027 | 0.013 |
| MCF7 | 2.917 | 1.309 |
| ZR75-1 | 9.771 | 0.229 |
| MDAMB415 | 5.290 | 0.536 |

Table S2. IC50 values were calculated from Fig. 1f.

| **Cell line** | **Treatment** | **IC50 (Mean, µM)** | **SEM** |
| --- | --- | --- | --- |
| MCF7 | Cobimetinib | 8.891 | 0.810 |
|  | RSL3 | 1.839 | 0.117 |
|  | RSL3+Cobimetinib | 0.823 | 0.466 |

Table S3. IC50 values were calculated from Fig. 2a.

| **Cell line** | **Treatment** | **IC50 (Mean, µM)** | **SEM** |
| --- | --- | --- | --- |
| MDAMB415 | Neratinib | 10.000 | 0.000 |
|  | RSL3 | 5.789 | 0.506 |
|  | Neratinib+RSL3 | 0.444 | 0.010 |
|  | Lapatinib | 10.000 | 0.000 |
|  | RSL3 | 5.789 | 0.506 |
|  | Lapatinib+RSL3 | 8.433 | 0.082 |
|  | Gefitinib | 8.433 | 1.567 |
|  | RSL3 | 5.789 | 0.506 |
|  | Gefitinib+RSL3 | 3.996 | 0.195 |
| ZR75-1 | Neratinib | 10.000 | 0.000 |
|  | RSL3 | 5.342 | 0.685 |
|  | Neratinib+RSL3 | 1.660 | 0.357 |
|  | Lapatinib | 10.000 | 0.000 |
|  | RSL3 | 5.342 | 0.685 |
|  | Lapatinib+RSL3 | 4.187 | 0.128 |
|  | Gefitinib | 10.000 | 0.000 |
|  | RSL3 | 5.342 | 0.685 |
|  | Gefitinib+RSL3 | 3.965 | 0.692 |
| MCF7 | Neratinib | 2.147 | 1.427 |
|  | RSL3 | 2.381 | 0.675 |
|  | Neratinib+RSL3 | 0.498 | 0.324 |
|  | Lapatinib | 9.839 | 0.267 |
|  | RSL3 | 2.381 | 0.675 |
|  | Lapatinib+RSL3 | 1.632 | 0.397 |
|  | Gefitinib | 10.000 | 0.000 |
|  | RSL3 | 2.381 | 0.675 |
|  | Gefitinib+RSL3 | 2.270 | 0.797 |

Table S4. IC50 values were calculated from Supplementary Fig. 2a

| **Cell line** | **Treatment** | **IC50 (Mean, µM)** | **SEM** |
| --- | --- | --- | --- |
| MDAMB415 | Afatinib | 4.675 | 0.465 |
|  | RSL3 | 1.900 | 1.307 |
|  | Afatinib+RSL3 | 0.543 | 0.416 |
|  | Dacomitinib | 4.803 | 0.870 |
|  | RSL3 | 1.900 | 1.307 |
|  | Dacomitinib+RSL3 | 0.515 | 0.395 |
|  | Sapitinib | 10.000 | 0.000 |
|  | RSL3 | 5.789 | 0.506 |
|  | Sapitinib+RSL3 | 4.788 | 0.195 |
|  | Tucatinib | 10.000 | 0.000 |
|  | RSL3 | 1.608 | 1.452 |
|  | Tucatinib+RSL3 | 1.457 | 1.398 |
| MCF7 | Afatinib | 1.834 | 0.302 |
|  | RSL3 | 2.088 | 0.849 |
|  | Afatinib+RSL3 | 0.997 | 0.243 |
|  | Dacomitinib | 2.247 | 0.181 |
|  | RSL3 | 2.381 | 0.675 |
|  | Dacomitinib+RSL3 | 1.033 | 0.166 |
|  | Sapitinib | 10.000 | 0.000 |
|  | RSL3 | 2.388 | 0.664 |
|  | Sapitinib+RSL3 | 1.525 | 0.607 |
|  | Tucatinib | 10.000 | 0.000 |
|  | RSL3 | 2.088 | 0.849 |
|  | Tucatinib+RSL3 | 2.477 | 0.380 |

Table S5. IC50 values were calculated from Supplementary Fig. 2b.

| **Cell line** | **Treatment** | **IC50 (Mean, µM)** | **SEM** |
| --- | --- | --- | --- |
| MDAMB415 | Neratinib | 10.000 | 0.000 |
|  | Erastin | 10.000 | 1.057 |
|  | Neratinib+Erastin | 10.000 | 0.000 |
| MCF7 | Neratinib | 3.813 | 3.094 |
|  | Erastin | 10.000 | 0.330 |
|  | Neratinib+Erastin | 3.534 | 3.233 |
| ZR75-1 | Neratinib | 10.000 | 0.000 |
|  | Erastin | 10.000 | 0.000 |
|  | Neratinib+Erastin | 10.000 | 0.000 |
| MDAMB175VII | Neratinib | 0.046 | 0.046 |
|  | Erastin | 1.371 | 0.346 |
|  | Neratinib+Erastin | 0.472 | 0.152 |
| MDAMB453 | Neratinib | 0.314 | 0.294 |
|  | Erastin | 6.934 | 3.066 |
|  | Neratinib+Erastin | 0.930 | 0.443 |

Table S6. IC50 values were calculated from Fig. 2b.

| **Cell line** | **Treatment** | **IC50 (Mean, µM)** | **SEM** |
| --- | --- | --- | --- |
| MDAMB415 | Negative shRNA_Neratinib | 4.834 | 0.367 |
|  | GPX4 shRNA#4_Neratinib | 0.493 | 0.057 |
|  | GPX4 shRNA#5_Neratinib | 0.947 | 0.349 |
| MCF7 | Negative shRNA_Neratinib | 5.431 | 1.981 |
|  | GPX4 shRNA#4_Neratinib | 0.547 | 0.271 |
|  | GPX4 shRNA#5_Neratinib | 1.714 | 0.788 |

Table S7. IC50 values were calculated from Supplementary Fig. 2f.

| **Cell line** | **Treatment** | **IC50 (Mean, µM)** | **SEM** |
| --- | --- | --- | --- |
| MDAMB415 | ML210 | 10.000 | 0.000 |
|  | Neratinib | 5.707 | 0.950 |
|  | ML210+Neratinib | 0.705 | 0.169 |
| MCF7 | ML210 | 10.000 | 0.000 |
|  | Neratinib | 6.163 | 2.130 |
|  | ML210+Neratinib | 2.987 | 1.715 |

Table S8. IC50 values were calculated from Fig. 2e.

| **Cell line** | **Type** | **IC50 (RSL3, Mean, µM)** | **SEM** |
| --- | --- | --- | --- |
| BT474 | Parent cell | 0.059 | 0.009 |
|  | Resistant cell | 0.101 | 0.016 |
|  | Recovery cell | 0.062 | 0.007 |
